# COVIDiSTRESS diverse dataset on psychological and behavioural outcomes one year into the COVID-19 pandemic

**DOI:** 10.1038/s41597-022-01383-6

**Published:** 2022-06-21

**Authors:** Angélique M. Blackburn, Sara Vestergren, the COVIDiSTRESS II Consortium, Angélique M. Blackburn, Sara Vestergren, Thao P. Tran, Sabrina Stöckli, Siobhán M. Griffin, Evangelos Ntontis, Alma Jeftic, Stavroula Chrona, Gözde Ikizer, Hyemin Han, Taciano L. Milfont, Douglas Parry, Grace Byrne, Mercedes Gómez-López, Alida Acosta, Marta Kowal, Gabriel De Leon, Aranza Gallegos, Miles Perez, Mohamed Abdelrahman, Elayne Ahern, Ahmad Wali Ahmad Yar, Oli Ahmed, Nael H. Alami, Rizwana Amin, Lykke E. Andersen, Bráulio Oliveira Araújo, Norah Aziamin Asongu, Fabian Bartsch, Jozef Bavoľár, Khem Raj Bhatta, Tuba Bircan, Shalani Bita, Hasitha Bombuwala, Tymofii Brik, Huseyin Cakal, Marjolein Caniëls, Marcela Carballo, Nathalia M. Carvalho, Laura Cely, Sophie Chang, Maria Chayinska, Fang-Yu Chen, Brendan Ch’ng, JohnBosco Chika Chukwuorji, Ana Raquel Costa, Vidijah Ligalaba Dalizu, Eliane Deschrijver, İlknur Dilekler Aldemir, Anne M. Doherty, Rianne Doller, Dmitrii Dubrov, Salem Elegbede, Jefferson Elizalde, Eda Ermagan-Caglar, Regina Fernández-Morales, Juan Diego García-Castro, Rebekah Gelpí, Shagofah Ghafori, Ximena Goldberg, Catalina González-Uribe, Harlen Alpízar-Rojas, Christian Andres Palacios Haugestad, Diana Higuera, Kristof Hoorelbeke, Evgeniya Hristova, Barbora Hubená, Hamidul Huq, Keiko Ihaya, Gosith Jayathilake, Enyi Jen, Amaani Jinadasa, Jelena Joksimovic, Pavol Kačmár, Veselina Kadreva, Kalina Kalinova, Huda Anter Abdallah Kandeel, Blerina Kellezi, Sammyh Khan, Maria Kontogianni, Karolina Koszałkowska, Krzysztof Hanusz, David Lacko, Miguel Landa-Blanco, Yookyung Lee, Andreas Lieberoth, Samuel Lins, Liudmila Liutsko, Amanda Londero-Santos, Anne Lundahl Mauritsen, María Andrée Maegli, Patience Magidie, Roji Maharjan, Tsvetelina Makaveeva, Malose Makhubela, María Gálvis Malagón, Sergey Malykh, Salomé Mamede, Samuel Mandillah, Mohammad Sabbir Mansoor, Silvia Mari, Inmaculada Marín-López, Tiago A. Marot, Sandra Martínez, Juma Mauka, Sigrun Marie Moss, Asia Mushtaq, Arian Musliu, Daniel Mususa, Arooj Najmussaqib, Aishath Nasheeda, Ramona Nasr, Natalia Niño Machado, Jean Carlos Natividade, Honest Prosper Ngowi, Carolyne Nyarangi, Charles Ogunbode, Charles Onyutha, K. Padmakumar, Walter Paniagua, Maria Caridad Pena, Martin Pírko, Mayda Portela, Hamidreza Pouretemad, Nikolay Rachev, Muhamad Ratodi, Jason Reifler, Saeid Sadeghi, Harishanth Samuel Sahayanathan, Eva Sanchez, Ella Marie Sandbakken, Dhakal Sandesh, Shrestha Sanjesh, Jana Schrötter, Sabarjah Shanthakumar, Pilleriin Sikka, Konstantina Slaveykova, Anna Studzinska, Fadelia Deby Subandi, Namita Subedi, Gavin Brent Sullivan, Benjamin Tag, Takem Ebangha Agbor Delphine, William Tamayo-Agudelo, Giovanni A. Travaglino, Jarno Tuominen, Tuğba Türk-Kurtça, Matutu Vakai, Tatiana Volkodav, Austin Horng-En Wang Wang, Alphonsus Williams, Charles Wu, Yuki Yamada, Teodora Yaneva, Nicolás Yañez, Yao-Yuan Yeh, Emina Zoletic

**Affiliations:** 1 Texas A&M International University, Laredo, TX USA; 2 Keele University, Keele, England; 3 Department of Psychology and Communications, Texas A&M International University, Laredo, Texas USA; 4 Keele University, Keele, UK; 5 Colorado State University, Fort Collins, Colorado, USA; 6 University of Bern, Bern, Switzerland; 7 Department of Psychology, University of Limerick, Limerick, Ireland; 8 Canterbury Christ Church University, UK & The Open University, Milton Keynes, UK; 9 Peace Research Institute, International Christian University, Tokyo, Japan; 10 Queen’s University Belfast, School of History, Anthropology, Belfast, Northern Ireland; 11 TOBB University of Economics and Technology, Ankara, Turkey; 12 University of Alabama, Tuscaloosa, USA; 13 School of Psychology, University of Waikato, Hamilton, New Zealand; 14 Department of Information Science, Stellenbosch University, Stellenbosch, South Africa; 15 Vrije Universiteit Amsterdam, Amsterdam, Netherlands; 16 Universidad de Córdoba, Córdoba, Spain; 17 Faculty of Health, Universidad Autónoma de Bucaramanga, Bucaramanga, Colombia; 18 Institute of Psychology, University of Wroclaw, Wroclaw, Poland; 19 Texas A&M International University, Laredo, Texas USA; 20 Doha Institute for Graduate Studies, Doha, Qatar; 21 Dublin City University, Dublin, Ireland; 22 Vrije Universiteit Brussel, Brussels, Belgium; 23 University of Chittagong, Chittagong, Bangladesh; 24 Modern University for Business and Science, Beirut, Lebanon; 25 Bahria University Islamabad Campus, Islamabad, Pakistan; 26 Sustainable Development Solutions Network - Bolivia, La Paz, Bolivia; 27 University of Porto, Porto, Portugal; 28 National Centre for Education, Yaounde, Cameroon; 29 Montpellier Buisness School, Montpellier, France; 30 Department of psychology, Faculty of Arts, Pavol Jozef Safarik University in Kosice, Kosice, Slovakia; 31 Master’s program in Counseling Psychology, Tribhuvan University, Kathmandu, Nepal; 32 Tarbiat Modares University, Teheran, Iran; 33 Icare Sustainably international, International, Switzerland; 34 Kyiv School of Economics, Kyiv, Ukraine; 35 Open Universiteit, Heerlen, Netherlands; 36 Departamento de Neurociencia y Aprendizaje, Universidad Católica del Uruguay, Montevideo, Uruguay; 37 Pontifical Catholic University of Rio de Janeiro, Rio de Janeiro, Brazil; 38 Universidad de Los Andes, Bogotá, Colombia; 39 Radboud University, Nijmegen, Netherlands; 40 Pontificia Universidad Católica de Chile, Santiago, Chile; 41 Soochow University, Taiwan, Taipei Taiwan; 42 University of Malaya, Kuala Lumpur, Malaysia; 43 University of Nigeria, Nsukka, Nigeria; 44 The Hill School, Eldoret, Kenya; 45 Ghent University, Ghent, Belgium; 46 University of New South Wales, Kensington, Australia; 47 University College Dublin, Dublin, Ireland; 48 National Research University Higher School of Economics, Moscow, Russian Federation; 49 Universidad del Azuay, Cuenca, Ecuador; 50 The Embassy of Turkey in North Nicosia, Nicosia, Cyprus; 51 Universidad Francisco Marroquin, Guatemala City, Guatemala; 52 Universidad de Costa Rica, Sede de Occidente, Costa Rica, San Ramón, Costa Rica; 53 University of Toronto, Toronto, Canada; 54 ISGlobal (Barcelona Institute for Global Health), Barcelona, Spain; 55 University of Oslo, Norway, Oslo Norway; 56 New Bulgarian University, Sofia, Bulgaria; 57 Ministry of Health, Prague, Czechia; 58 United International University, Dhaka, Bangladesh; 59 Fukuoka Institude Technology, Fukuoka, Japan; 60 University of Belgrade, Faculty of philosophy, Department of psychology, Belgrade, Serbia; 61 Leiden University, Leiden, Netherlands; 62 Department of psychology, Faculty of Arts, Assiut University in Egypt, Asyut, Egypt; 63 Nottingham Trent University, Nottingham, UK; 64 Örebro University, Örebro, Sweden; 65 University of Lodz, Poland, Institute of Psychology, Lodz, Poland; 66 Institute of Psychology Polish Academy of Sciences, Warszawa, Poland; 67 Masaryk University, Brno, Czechia; 68 School of Psychological Sciences, National Autonomous University of Honduras (UNAH), Tegucigalpa, Honduras; 69 The University of Texas at Austin, Austin, Texas USA; 70 Aarhus University, Aarhus, Denmark; 71 Lomonosov MSU, Moscow, Russia; 72 URFU, Ekaterinburg, Russia; 73 Federal University of Rio de Janeiro, Rio de Janeiro, Brazil; 74 Independent Researcher, South Africa, South Africa; 75 Tribhuvan University, Kathmandu, Nepal; 76 Sofia University St. Kliment Ohridski, Sofia, Bulgaria; 77 University of Limpopo, Department of Psychology, Polokwane, South Africa; 78 Russian Academy of Education, Moscow, Russia; 79 AIC RAISE Business Incubator, Rathinam College of Arts and Science India, Coimbatore, India; 80 Trichandra Multiple Campus, Tribhuvan University, Kathmandu, Nepal; 81 University of Milano-Bicocca, Milan, Italy; 82 Rolffortress communications, Kenya, Kenya; 83 National University of Modern Languages, Islamabad, Pakistan; 84 Ludwig Maximilian University, Munich, Germany; 85 SIVIO Institute, Harare, Zimbabwe; 86 Villa College, Maldives, Malé Maldives; 87 Mzumbe University, Dar Es Salaam, Tanzania; 88 University of Nottingham, Nottingham, UK; 89 Department of Civil and Environmental Engineering, Kyambogo University, Uganda, Kampala Uganda; 90 Manipal Institute of Communication, Manipal Academy of Higher Education, Manipal, India; 91 Universidad Rafael Landivar, Guatemala City, Guatemala; 92 Universidad de Las Américas (UDLA), Quito, Ecuador; 93 Mendel University in Brno, Brno, Czechia; 94 Departamento de Psicologia, Universidad Católica de Uruguay, Montevideo, Uruguay; 95 Institute for Cognitive and Brain Sciences Shahid Beheshti University, Tehran, Iran; 96 Faculty of Health and Psychology, State Islamic University of Sunan Ampel, Surabaya, Indonesia; 97 University of Exeter, Exeter, UK; 98 Department of Finance University of Kelaniya, Colombo, Sri Lanka; 99 Oslo New University College, Oslo, Norway; 100 Pavol Jozef Safarik University in Kosice, Kosice, Slovakia; 101 University of Turku, Turku, Finland; 102 University of Skövde, Skövde, Sweden; 103 University of Wellington, Wellington, New Zealand; 104 ICAM, Toulouse, Tolouse France; 105 Sustainable Development Goals Hub, Universitas Indonesia, Depok, Indonesia; 106 International Psychoanalytic University Berlin, Berlin, Germany; 107 University of Melbourne, Melbourne, Australia; 108 National Institute of Cartography Cameroon, Yaounde, Cameroon; 109 Universidad Cooperativa de Colombia, Bogotá, Colombia; 110 Royal Holloway, University of London, London, UK; 111 Univeristy of Turku, Turku, Finland; 112 Trakya University, Edirne, Turkey; 113 North - West University, Potchefstroom, South Africa; 114 Kuban State University, Krasnodar, Russia; 115 University of Nevada, Las Vegas, USA; 116 Sustainable Environmental Solutions-Sweden, Uppsala, Sweden; 117 Purdue University, West Lafayette, Indiana, USA; 118 Kyushu University, Fukuoka, Japan; 119 University of St. Thomas, Houston, USA; 120 Doctoral School of Social Sciencies, University of Warsaw, Warsaw, Poland

**Keywords:** Health policy, Epidemiology, Human behaviour, Developing world, Research data

## Abstract

During the onset of the COVID-19 pandemic, the COVIDiSTRESS Consortium launched an open-access global survey to understand and improve individuals’ experiences related to the crisis. A year later, we extended this line of research by launching a new survey to address the dynamic landscape of the pandemic. This survey was released with the goal of addressing diversity, equity, and inclusion by working with over 150 researchers across the globe who collected data in 48 languages and dialects across 137 countries. The resulting cleaned dataset described here includes 15,740 of over 20,000 responses. The dataset allows cross-cultural study of psychological wellbeing and behaviours a year into the pandemic. It includes measures of stress, resilience, vaccine attitudes, trust in government and scientists, compliance, and information acquisition and misperceptions regarding COVID-19. Open-access raw and cleaned datasets with computed scores are available. Just as our initial COVIDiSTRESS dataset has facilitated government policy decisions regarding health crises, this dataset can be used by researchers and policy makers to inform research, decisions, and policy.

## Abstract (summary)

**Table:** 

Measurement(s)	demographics • social status • identity • perceived stress • loneliness • stressors • support • compliance • social norms • vaccine attitude • vaccine willingness • trust • resilience • conspiratorial thinking • anti-expert sentiment scale (AESS) • moral foundations • emotional regulation
Technology Type(s)	survey

## Background & Summary

The COVIDiSTRESS Global Survey (https://osf.io/2ftma/) was one of the largest studies regarding the global impact of COVID-19 during the initial months of the 2020 pandemic^1–3^. While other large-scale studies regarding the psychological impact of COVID-19 exist, most either focused on specific subsets of the population^4^ or specific countries^5–8^. The COVID-19 Global Survey was translated into 47 languages and administered in 179 countries. The Consortium generated a rich dataset that has resulted in a comprehensive understanding of the global effects of the pandemic^1,2^. The project highlighted not only the benefits of large-scale data collection using this method^9^, but also resulted in multiple publications and informed policy decisions within the first year^10,11^.

The current survey is an extension of the COVIDiSTRESS Consortium project to assess the global impact of COVID-19 approximately one year after the initial survey. This expands research from the initial COVIDiSTRESS Global Survey, in which we found that trust in government is linked to compliance with measures to reduce the impact of COVID-19^2^. Results from the COVIDiSTRESS study have corroborated other recent findings^12^. We used the same large-scale data collection methods as the initial survey. It was our goal to address questions that were left unanswered in the initial study and include countries that were not previously assessed.

**Fig. 1:**
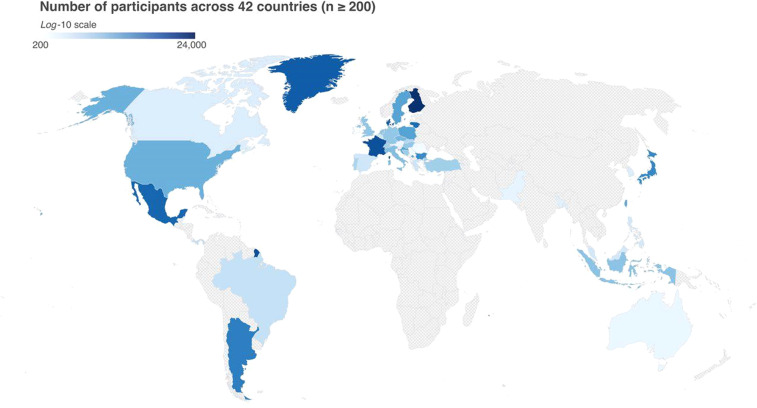
Map of data collected during the initial COVIDiSTRESS Global Survey. Only countries with more than 200 participants in the original survey are indicated. Image reproduced from Yamada *et al*. (2021)^1^, under the Creative Commons Attribution 4.0 International Licence.

One limitation of the initial study was the inability to collect sufficient data in certain regions. As can be seen in Fig. 1, although the first dataset had impressive global representation, less than 200 responses were received in Russia as well as most countries in Africa and Central Asia. Therefore, these regions became a priority for the second wave of data collection. The present survey for the dataset described here was released with the goals of addressing diversity, equity, and inclusion by working with a diverse group of over 150 researchers across the globe who collaborated to translate the survey into 48 languages and dialects and launched the survey locally in 137 countries.

In addition to questions about stress, loneliness, and trust in government, we added new items to the dataset to accommodate new policy developments, new information (and misinformation) about COVID-19, and attitudes about the newly released vaccines. Specifically, we collected demographic information and assessed social norms, compliance behaviours, vaccine hesitancy and attitudes, individuals’ stress and resilience, trust in scientists and the healthcare systems, moral values, and information acquisition and misperceptions regarding COVID-19.

This is a large-scale project with multiple hypotheses. Here we describe only the methods and details about the open-access dataset, available through the Open Science Framework. Specific hypotheses and analyses using the survey data will appear in separate publications.

## Methods

### Participants

**Fig. 2:**
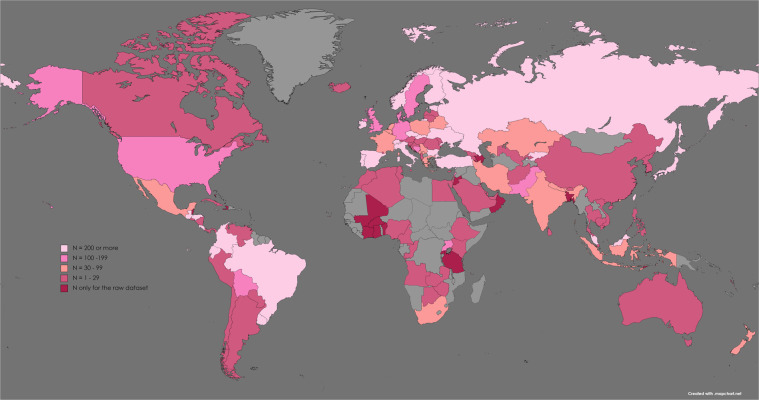
Map of data collected for the COVIDiSTRESS II Dataset (N = 15,740). Light pink: Countries with 200 or more participants in the cleaned dataset. Medium Pink: Countries with 100 or more, but less than 200 participants in the cleaned dataset. Salmon: Countries with 30 or more, but less than 100 participants in the cleaned dataset. Dark Pink: Countries with less than 30 participants in the cleaned dataset. Dark Red: Countries only with participants in the raw dataset. Note that small countries may not be represented. Map was created by Angélique Blackburn using mapchart.net, an open-access site created by Minas under the Creative Commons Attribution-ShareAlike 4.0 International License, and is published under the same license as the original work.

A total of 20,601 people from 137 countries accessed an online survey link to respond to questions about their experience with COVID-19 during the summer of 2021. After data cleaning, 15,740 individuals met the inclusion criteria: provided informed consent, 18 or more years of age, passed the attention check, and did not complete the entire survey in under three minutes. The countries represented in the cleaned and raw datasets are portrayed in Fig. 2. For convenience, demographic characteristics for countries with over 200 responses remaining in the cleaned dataset are presented in Table 1.

**Table 1:** Number of subjects by country and missing data.

Residing Country	N	Mean % Complete	N 50% Data	N 90% Data	N 1st Half	N 2nd Half	Prop_50%	Prop_90%	Prop_1st Half	Prop_2nd Half
Global	15740	91.5	15356	12991	13322	12956	97.6	82.5	84.6	82.3
**Russian Federation**	**2260**	89.9	2238	1784	1466	1784	99.0	78.9	64.9	78.9
**Japan**	**2133**	97.0	2126	2015	1929	2013	99.7	94.5	90.4	94.4
**Finland**	**963**	95.5	943	881	858	878	97.9	91.5	89.1	91.2
**Switzerland**	**593**	94.0	586	525	562	523	98.8	88.5	94.8	88.2
**Spain**	**575**	91.8	556	474	502	474	96.7	82.4	87.3	82.4
**Colombia**	**548**	91.0	523	443	453	438	95.4	80.8	82.7	79.9
**Portugal**	**484**	89.5	468	384	446	381	96.7	79.3	92.1	78.7
**Brazil**	**448**	93.7	443	390	420	389	98.9	87.1	93.8	86.8
**Honduras**	**429**	86.0	422	307	348	306	98.4	71.6	81.1	71.3
**Ireland**	**401**	88.2	374	306	342	302	93.3	76.3	85.3	75.3
**Norway**	**376**	93.3	365	324	325	319	97.1	86.2	86.4	84.8
**Czech Republic**	**365**	90.9	348	296	321	296	95.3	81.1	87.9	81.1
**Slovakia**	**313**	92.1	302	266	269	265	96.5	85.0	85.9	84.7
**Italy**	**310**	92.8	302	266	270	266	97.4	85.8	87.1	85.8
**Bulgaria**	**299**	93.1	292	256	264	258	97.7	85.6	88.3	86.3
**Ecuador**	**291**	87.3	282	210	257	208	96.9	72.2	88.3	71.5
**Uruguay**	**288**	89.4	280	218	241	213	97.2	75.7	83.7	74.0
**Guatemala**	**287**	89.4	278	221	246	217	96.9	77.0	85.7	75.6
**Costa Rica**	**270**	91.4	266	217	241	217	98.5	80.4	89.3	80.4
**Kyrgyzstan**	**254**	87.0	238	185	220	189	93.7	72.8	86.6	74.4
**Ukraine**	**252**	93.4	249	212	215	209	98.8	84.1	85.3	82.9
**Estonia**	**246**	92.4	236	206	198	206	95.9	83.7	80.5	83.7
**Malaysia**	**225**	87.0	216	167	194	167	96.0	74.2	86.2	74.2
**Taiwan**	**221**	94.9	220	197	203	197	99.5	89.1	91.9	89.1
**Turkey**	**200**	85.4	187	145	167	145	93.5	72.5	83.5	72.5
*Pakistan*	*157*	82.9	152	101	148	103	96.8	64.3	94.3	65.6
*Germany*	*152*	92.7	147	130	137	128	96.7	85.5	90.1	84.2
*Lebanon*	*141*	85.4	134	97	125	97	95.0	68.8	88.7	68.8
*Uganda*	*135*	92.9	134	118	130	120	99.3	87.4	96.3	88.9
*Sweden*	*134*	92.6	131	110	115	109	97.8	82.1	85.8	81.3
*United Kingdom of Great Britain and Northern Ireland*	*134*	93.8	134	116	121	115	100.0	86.6	90.3	85.8
*Denmark*	*127*	89.8	119	98	108	96	93.7	77.2	85.0	75.6
*Bolivia*	*115*	89.7	111	90	97	88	96.5	78.3	84.3	76.5
*United States of America*	*114*	91.1	111	93	103	93	97.4	81.6	90.4	81.6
*Bosnia and Herzegovina*	*109*	86.0	102	75	89	74	93.6	68.8	81.7	67.9
*Iran, Islamic Republic of…*	*90*	92.2	88	76	76	76	97.8	84.4	84.4	84.4
*India*	*88*	88.0	87	65	78	65	98.9	73.9	88.6	73.9
*Poland*	*87*	89.0	85	65	78	64	97.7	74.7	89.7	73.6
*Mexico*	*83*	96.2	82	78	80	77	98.8	94.0	96.4	92.8
*Greece*	*54*	90.7	53	43	48	43	98.1	79.6	88.9	79.6
*Indonesia*	*49*	90.0	47	39	45	39	95.9	79.6	91.8	79.6
*Kosovo*	*48*	83.8	46	31	41	31	95.8	64.6	85.4	64.6
*Nepal*	*44*	88.9	42	34	39	35	95.5	77.3	88.6	79.5
*South Africa*	*44*	89.5	44	33	37	32	100.0	75.0	84.1	72.7
*Hong Kong (S.A.R.)*	*40*	83.5	40	24	34	24	100.0	60.0	85.0	60.0
*Maldives*	*39*	89.6	35	32	33	32	89.7	82.1	84.6	82.1
*New Zealand*	*38*	91.0	36	31	33	31	94.7	81.6	86.8	81.6
*Kazakhstan*	*36*	90.2	36	28	29	28	100.0	77.8	80.6	77.8
*Montenegro*	*35*	79.4	31	20	26	19	88.6	57.1	74.3	54.3
*Netherlands*	*35*	93.6	34	31	32	30	97.1	88.6	91.4	85.7
*Serbia*	*35*	82.6	34	21	31	21	97.1	60.0	88.6	60.0
*Belarus*	*34*	90.7	33	26	29	27	97.1	76.5	85.3	79.4
*Belgium*	*34*	93.6	34	29	29	28	100.0	85.3	85.3	82.4
*France*	*32*	89.4	31	25	26	25	96.9	78.1	81.3	78.1
*Other*	*446*									

Response rates in the cleaned dataset are provided for countries with more than 200 participants (in **bold**) and 30 participants (in *italic*). Note that the cleaned dataset has excluded any participants who failed the attention check or did not otherwise qualify for inclusion. Because the survey was presented in two parts, the number of participants who completed each part are also presented, along with the average percentage of data completion by country. Abbreviations: Mean % Complete = average percentage of survey complete across all subjects. N 50% Data = Number of subjects for whom 50% of the data is complete. N 90% Data = Number of subjects for whom 50% of the data is complete. N 1st Half = Number of subjects who completed the first half of the survey. N 2nd Half Number of subjects who completed the second half of the survey. Prop50% = Proportion of subjects for whom 50% of the data is available. Prop90% = Proportion of subjects for whom 50% of the data is available. Prop 1st Half = Proportion of subjects who completed the first half. Prop 2nd Half = Proportion of subjects who completed the second half.

Participants were recruited through concentrated local efforts by a team of over 150 international researchers, including word of mouth, press releases, TV, email lists, and social media. Data was collected anonymously, and participants volunteered without monetary compensation. All participants reported being over the age of 18. Demographic data, including responses by country, will be discussed below and can be found in Tables 1–5. For ease of comparison, population equivalents have been provided in these tables aside demographic data for the population in this study.

**Table 2:** Age and gender of participants overall and across countries with more than 200 participants.

Residing Country	Age *M* (years)	Age *SD* (years)	Prop_Female (%)	Pop_Age (Median)	Pop_Female (%)
All Countries	36.4	14.3	67.1	30.9	49.6
Russian Federation	26.1	10.5	70.9	39.6	53.7
Japan	45.5	11.1	41.9	48.4	51.2
Finland	46.1	14.4	78.4	43.1	50.7
Switzerland	44.8	19.0	63.6	43.1	50.4
Spain	40.4	13.8	64.5	44.9	50.9
Colombia	40.0	12.6	67.9	31.3	50.9
Portugal	33.3	14.9	70.2	46.2	52.7
Brazil	38.6	13.2	72.3	33.5	50.9
Honduras	25.9	8.1	66.9	24.3	50.0
Ireland	29.0	10.8	67.8	38.2	50.4
Norway	40.9	13.6	80.3	39.8	49.5
Czech Republic	34.1	11.3	70.7	43.2	50.8
Slovakia	34.5	13.6	88.8	41.2	51.3
Italy	44.8	16.3	74.2	47.3	51.3
Bulgaria	41.5	16.7	73.6	44.6	51.4
Ecuador	31.8	10.8	66.3	27.9	50.0
Uruguay	42.0	12.9	87.8	35.8	51.7
Guatemala	36.9	14.0	84.3	22.9	50.7
Costa Rica	35.9	10.4	69.6	33.5	50.0
Kyrgyzstan	32.4	12.5	82.3	26.0	50.5
Ukraine	31.4	10.1	63.5	41.2	53.7
Estonia	39.4	10.2	86.2	42.4	52.6
Malaysia	27.2	8.8	69.3	30.3	48.6
Taiwan	34.9	9.8	62.0	42.5	–
Turkey	23.8	8.3	68.5	31.5	50.6

Abbreviations: Age *M* = mean age in years of participants in this study. Age *SD = *standard deviation of Age. Prop_female = percentage of women in this study compared to other genders. Pop_Age = United Nations projections of 2020 median age of the population equivalent, under Creative Commons license CC BY 3.0 (http://creativecommons.org/licenses/by/3.0/igo/)^39^. Pop_Female = population equivalent for percentage of population that is female according to The World Bank: Population, female (% of total population): based on age/sex distributions of United Nations Population Division’s World Population Prospects: 2019 Revision., under Creative Commons Attribution CC BY 4.0 (https://datacatalog.worldbank.org/public-licenses#cc-by)^40^.

**Table 3:** Education background overall and across countries with more than 200 participants.

Residing Country	12years + (%)	Uni Degree (%)	PhD (%)	Pop_ Secondary (%)	Pop_ Tertiary (%)
All Countries	41.5	48.6	6.4	–	–
Russian Federation	66.2	28.9	0.7	85.0	24.7
Japan	57.2	31.7	1.0	80.3	18.9
Finland	35.1	54.7	4.7	77.5	11.9
Switzerland	42.5	45.4	6.9	86.6	17.9
Spain	24.7	53.9	20.7	53.3	15.0
Colombia	15.7	77.0	5.7	53.2	18.6
Portugal	33.1	42.6	23.6	43.5	3.3
Brazil	18.8	66.1	15.2	47.4	5.6
Honduras	76.2	20.0	1.4	29.9	1.9
Ireland	46.4	48.4	4.7	70.8	26.8
Norway	27.9	61.2	8.2	78.7	12.2
Czech Republic	47.9	46.0	5.8	90.7	7.6
Slovakia	39.6	48.6	8.0	87.7	8.8
Italy	44.8	45.2	6.5	52.5	6.8
Bulgaria	42.8	50.5	6.0	77.8	13.1
Ecuador	26.1	69.8	2.7	43.4	5.2
Uruguay	18.4	74.3	5.9	31.5	3.5
Guatemala	28.6	65.9	4.9	24.0	0.0
Costa Rica	18.5	78.1	1.1	40.9	14.7
Kyrgyzstan	41.7	52.4	2.8	88.4	9.0
Ukraine	11.1	77.4	10.3	74.3	24.6
Estonia	40.2	56.1	1.2	85.8	18.9
Malaysia	41.8	53.8	4.4	62.6	5.8
Taiwan	5.4	89.1	5.4	–	8.2
Turkey	62.0	34.5	3.0	42.2	5.3

Note that full dataset contains additional categories. Abbreviations: 12years +  = percentage of participants that have at least 12 years of education; collapsed across 12 years and some university. Uni_Degree = percentage of participants who have bachelor’s or master’s degrees. PhD = percentage of participants who have PhD. Pop_Secondary (%) = population equivalent of proportion of population aged 25 + who have completed at least upper secondary education; reflects 2001–2021 data sourced from UNESCO Institute for Statistics (uis.unesco.org) as of September 2021 and reported by The World Bank: Educational attainment, at least completed upper secondary, population 25 + , total (%) (cumulative), under Creative Commons Attribution CC BY 4.0 (https://datacatalog.worldbank.org/public-licenses#cc-by)^41^. Pop_Tertiary (%) = population equivalent of percentage of population aged 15 + who have completed tertiary education; reflects 2010 data sourced from Barro-Lee Data (1950–2010), http://www.barrolee.com/, updated September 2021^42^.

**Table 4:** Marital status of participants overall and across countries with more than 200 participants.

Residing Country	Single (%)	Dating (%)	Married (%)	Cohabitating (%)	Sep/Divorced (%)	Widowed (%)	Pop_Married (per 1000 habitants)
**All Countries**	31.9	15.0	33.5	11.9	4.6	1.0	–
Russian Federation	34.9	23.1	23.3	9.2	4.4	0.6	9.2
Japan	35.3	5.3	50.2	1.1	4.7	0.9	4.9
Finland	19.6	7.0	42.7	20.4	7.5	1.6	4.8
Switzerland	21.4	4.6	33.7	33.7	3.2	1.9	4.8
Spain	25.4	16.3	34.4	17.6	5.0	0.9	3.7
Colombia	29.6	15.0	31.6	15.7	7.1	0.7	2.2
Portugal	44.6	23.8	20.5	7.0	2.9	1.2	3.3
Brazil	28.8	16.7	36.2	10.0	7.1	0.7	4.7
Honduras	45.7	30.1	11.7	8.9	2.1	0.2	2.6
Ireland	44.1	27.7	15.7	9.5	2.5	0.2	4.6
Norway	14.6	9.0	35.1	32.2	5.1	0.8	4.4
Czech Republic	22.2	14.8	35.1	21.9	3.0	1.1	5.0
Slovakia	26.5	23.0	32.6	8.3	8.0	0.3	5.8
Italy	24.2	17.1	34.8	10.0	8.4	2.3	3.2
Bulgaria	17.4	18.1	33.4	17.7	5.4	5.0	4.0
Ecuador	40.9	18.2	26.5	7.9	5.8	0.7	5.6
Uruguay	15.3	11.8	39.6	19.8	10.1	2.8	3.7
Guatemala	26.8	16.4	41.5	8.0	5.9	0.7	4.4
Costa Rica	30.0	20.0	25.6	19.6	3.7	0.7	5.2
Kyrgyzstan	28.3	9.4	42.9	3.9	5.5	1.6	8.4
Ukraine	27.8	12.7	39.3	11.5	6.7	0.4	5.9
Estonia	18.7	9.3	34.6	31.3	4.1	1.2	4.9
Malaysia	64.9	14.7	16.4	0.9	0.9	0.4	–
Taiwan	43.0	15.8	31.7	7.7	1.4	0.0	–
Turkey	61.0	24.0	8.0	3.0	0.5	1.0	7.1

Abbreviations: Single = proportion of single participants. Dating = proportion of participants who are dating. Married = proportion of married participants. Cohabiting = proportion of participants who are cohabiting. Sep/Divorced = proportion of participants who are separated or divorced. Widowed = proportion of participants who are widowed. Note that full dataset contains additional categories. Pop_Married = for comparison, this variable includes the yearly marriage rate (variable dates from 1986–2018) per 1,000 people in the equivalent population, using data compiled from the Eurostat dataset (https://ec.europa.eu/eurostat/statistics-explained/index.php?title = Marriage_and_divorce_statistics#Fewer_marriages.2C_more_divorces), the OECD Family Database (https://www.oecd.org/els/family/database.htm), and the UN World Marriage Database (https://www.un.org/en/development/desa/population/publications/dataset/marriage/data.asp), under Creative Commons Attribution CC BY 4.0 (https://creativecommons.org/licenses/by/4.0/)^43^.

**Table 5:** COVID-19 history of participants across countries with more than 200 participants.

Residing Country	COVID_No (%)	COVID_Yes (%)	COVID_Unsure (%)	Cumulative Cases (per 1 M)
**All Countries**	63.0	21.7	15.3	–
Russian Federation	36.4	37.2	26.3	45599.4
Japan	94.6	2.5	2.9	10380.1
Finland	81.8	6.0	12.1	22090.9
Switzerland	66.9	13.3	19.7	86370.4
Spain	71.8	11.5	16.7	102052.2
Colombia	48.9	34.5	16.6	95376.1
Portugal	69.6	13.6	16.7	100258.4
Brazil	65.6	26.3	7.8	96148.8
Honduras	50.1	28.7	21.2	32478.4
Ireland	68.8	18.5	12.7	67654.7
Norway	81.6	9.8	8.5	27189.6
Czech Republic	45.5	36.4	18.1	156417.9
Slovakia	61.3	26.8	11.8	142854.0
Italy	64.2	20.3	15.5	74288.6
Bulgaria	40.1	34.4	25.1	63986.8
Ecuador	64.6	21.6	13.7	27877.1
Uruguay	78.8	11.8	9.4	110208.7
Guatemala	71.4	18.5	10.1	24068.8
Costa Rica	56.3	21.5	22.2	85744.8
Kyrgyzstan	20.1	59.8	20.1	26238.8
Ukraine	37.3	46.4	16.3	54481.8
Estonia	71.1	15.0	13.8	104745.1
Malaysia	82.7	1.3	16.0	47445.8
Taiwan	88.7	1.8	9.5	667.6
Turkey	63.5	19.0	17.5	73088.7

Participants were asked if they thought they had ever had COVID-19. Abbreviations: COVID_No = percentage of participants who did not think they had been infected with COVID-19. COVID_Yes = percentage of participants who thought they had been infected with COVID-19. COVID_Unsure = percentage of participants who did not know if they had been infected with COVID-19. Note that data was missing for 0.1% of respondents. Cumulative Cases = total cumulative COVID-19 cases on August 22, 2021 (last official date of data collection) per 1 M habitants, data originally sourced from COVID-19 Data Repository by the Center for Systems Science and Engineering (CSSE) at Johns Hopkins University (https://github.com/CSSEGISandData/COVID-19)^44^ and obtained from OldWorldinData.org Under Creative Commons Attribution CC BY 4.0 (https://creativecommons.org/licenses/by/4.0/)^45^.

### Materials

#### Survey overview

The full survey in English can be accessed directly at https://osf.io/az7s5/. The full list of variables included in the survey as well as the response options participants used to answer the survey are also available at OSF | COVIDiSTRESS Global Survey - Round II https://osf.io/36tsd/.

This survey contains a combination of validated scales and modified questions, each of which can be analyzed for relationships with other variables and across countries. The survey was divided into two sections: main variables presented to all participants at the beginning and optional variables in the second half. In this way, participants could opt to exit the survey after the main variables at the end of the first half or continue to the second half of the study. The survey also contained one attention check item to ensure that participants were paying attention.

For the greatest comparability across studies, some variables, the translation process, recruitment, and data collection procedures mirrored the method used for the initial COVIDiSTRESS Global Survey as closely as possible (see https://osf.io/2ftma/)^1^.

#### Variables

The survey contained individual items as well as previously validated, modified, and newly-designed scales. A full list of these variables and composite scales can be found with the data files. In brief, the first half of the survey contained demographic information including age, gender, residing country, birth country, education, occupation, work location, study location, relationship status, dependents, living situation with cohabiting adults and/or children, whether children were being home schooled, and socioeconomic status. It also contained single items regarding personal experience with COVID-19 and vaccine willingness, as well as the following scales: an adapted MacArthur Scale of Subjective Social Status (SSS-fam)^13,14^; Identity (4 items adapted from identity categories identified in previous research^15,16^) the Perceived Stress Scale (PSS-10; 10 items^17^), Loneliness Scale (SLON-3; 3 items as part of the extended PSS-10), Stressors (18 items, adapted from primary and secondary stress categories^18^), Perceived Support Scale (3 items adapted from a scale of perceived social support during a natural disaster^19^), Compliance (8 items adapted from surveys about compliance with measures to reduce the spread of the flu and pandemics^15,20^), Social Norms (16 items linked to the compliance scale), Vaccine Attitudes (6 items adapted from the Vaccine Attitude Question Battery^21^), and Trust in institutions including the government, health care, and science (7 items^1^). The second half of the survey included the Brief Resilience Scale (6 items^22^, the five item version of the Intolerance of Uncertainty Scale (IUS-5; 5 items^23^), an 8 item Scale of Information Acquisition Regarding COVID-19 adapted from previous research about popular sources of health information^24,25^, Misperceptions about COVID-19 (6 items created based on common misperceptions and studies of misperceptions^26^), the Conspiratorial Thinking Scale (4 items^26,27^), Anti-Expert Sentiment (3 items created by Consortium experts in the field based on previous research^26^), 11 items from the Moral Foundations Questionnaire^28,29^, and the Emotion Regulation Questionnaire (8 items^30^).

#### Ethical considerations and diversity, equity, and inclusion in survey creation

The Consortium conducted ethics meetings to ensure that survey questions were culturally and internationally inclusive. Our aim was to create an inclusive survey to capture a diverse population, including individuals from regions underrepresented in the original study. To protect participants and avoid sensitive or potentially damaging information collection, participants were not asked whether they had been diagnosed with COVID-19, whether they had been vaccinated, or other aspects of their medical status. In addition, care was taken during drafting of the survey to ensure that no questions about vaccine attitudes were written as leading questions or in ways that might influence vaccine attitudes. Finally, data collection was anonymous–we did not collect data that would allow identification of participants. Ethical approval for this study was obtained at the University of Salford (UK), as well as local ethical approval where required.

#### Translation

The survey was translated into 40 languages and adapted to the dialects of different regions, for a total of 48 versions. These languages and dialects with their codes in the related files are as follows: Afrikaans (AF), Arabic (AR), Bulgarian (BG), Bosnian (BS), Czech (CS), Danish (DA), Dari (DAR), German (DE), Greek (EL), English/American (EN-AM), Spanish-Bolivia (ES-BO), Spanish-Colombia (ES-CO), Spanish-Costa Rica (ES-CR), Spanish-Ecuador (ES-EC), Spanish-EU (ES-ES), Spanish-Guatemala (ES-G), Spanish-Honduras (ES-HN), Spanish-Mexico (ES-MX), Spanish-Uruguay (ES-UG), Estonian (ET), Farsi (FA), Finnish (FI), French (FR), Hindi (HI), Indonesian (ID), Italian (IT), Japanese (JA), Korean (KO), Nepali (NE), Dutch (NL), Norwegian (NO), Polish (PL), Portuguese (PT), Portuguese-Brazilian (PT-BR), Russian (RU), Sinhala (SIN), Slovak (SK), Albanian (SQI), Serbian (SR), Montenegrin (SR-ME), Swedish (SV), Swahili-Kenya (SW), Tamil (TA), Turkish (TR), Ukrainian (UK), Urdu (UR), Chinese - Simplified (ZH-S), Chinese - Traditional Taiwan (ZH-T). Translations were completed in teams following the three-step verification WHO method: forward translation from English, back-translation into English, and verification, as explained in the original study^9^. Whenever possible, a different translator performed each of the three steps.

### Data collection

Data was collected in Qualtrics. Links were generated for each language so researchers could use local recruitment methods to distribute the survey in the local language. The survey was launched online in multiple countries simultaneously, with rolling additions as the survey was translated into more languages.

The survey was available from the 10th of June to the 22^nd^ of August 2021, with the following extensions. Active data collection in Russia opened from May 28, 2021 through May 31, 2021, due to a need to collect the data in these countries before government restrictions regarding collection changed and active collection occurred in Uganda from May 29^th^, 2021 through June 30^th^, 2021 due to local team availability. Both Russia and Uganda were still open for participants throughout the main survey, however active data collection had ceased. Collection in Colombia and Sweden continued through August 29, 2021, for local ethical and team availability reasons. As such, the data is categorized as Russia/Uganda, Sweden/Colombia, and Main Dataset. All data was merged in the final dataset (https://osf.io/36tsd/).

## Data Records

### Data files

All data files can be found online at the Open Science Framework: OSF | COVIDiSTRESS Global Survey - Round II, under Final Data set [cleaned] COVIDiSTRESS II^31^. This folder contains a copy of the survey and author list. Along with a “Data used for cleaning” subfolder containing the three raw data files separated according to data collection dates and extensions (with corresponding files containing the numerical version of the data rather than choice text), we have provided a final cleaned data subfolder in which all raw data has been merged, invalid cases were excluded, and the data scales were re-coded. The first final cleaned file containing all data, “Final_COVIDiSTRESS_Vol2_Cleaned.csv,” is the primary file described herein; an additional file cleaned for SPSS is also contained in this folder. The R code used to clean the data is also available in the Codebook subfolder.

A separate folder for weekly data uploads comprises all raw data as collected each week throughout the study. The data collection registration files contain information about available translations and a detailed list of the measured variables with relevant notes about individual items and scale creation. Researchers may find it easiest to use the measured variables document in conjunction with the copy of the survey to obtain items for each variable of interest.

As most researchers will be interested in the Final COVIDiSTRESS Cleaned datafile, this file is described in more detail throughout this descriptor. This file contains the cleaned output of the Qualtrics survey with columns representing output in the order of the survey presentation, with additional columns at the end for calculated values as described in the Codebook and below. One row of data is available for each participant who was not excluded. It should be noted that all real, consenting participants are included in this file as long as they passed the attention check and participated for more than three minutes. Pilot subjects and excluded participants were removed as described below. Thus, while the technical validation performed here highlights countries with larger samples, researchers can access and use data for any valid participants based on their research design.

### Data cleaning

Both individual items and composite scores are present in the final cleaned dataset. Composite scores were calculated using the mean value for individual items. It should be noted that in some cases where validated scales were used, the scoring might differ from that in the original publications. In addition, use of composite scores is only justified once measurement invariance is achieved; while this information has been provided to allow researchers to determine useful variables for further analyses, further scale validation is critical.

Corrections were made to the raw dataset as follows:

- Data sets were combined to include those with extensions and the time zone was set to UTC. Columns were converted from character to numeric formats.
- Text responses were replaced with numeric values for Likert-type items.
- We filtered out cases without consent, test cases (100 cases), cases accessed through the preview link (4 cases), cases in which the respondent failed the attention check (1659 cases), and cases in which participants completed the survey in less than three minutes (but retained those who did not complete the survey).
- Data was recoded to align with the original scoring in previous studies. In particular, the Trust Scale was recoded from percentages to a 0–10 scale. The Perceived Stress Scale was recoded to a scale from 0–4.
- For the following items and scales, a neutral option was included on the survey: Vaccine Willingness, Identity, Perceived Support, Vaccine Attitudes, Emotions, and Moral Values. We created two versions for each of these scales and items–one with neutrals coded as 0 and the other with neutrals coded as the middle point of the response scale. Any composite scores were averaged after recoding individual items.
- “Not Applicable” (NA) responses to certain questions (numerical value = 99) were recoded in a separate column as NAppl (Not applicable) in order to store information about those who selected this option, as it is different from truly missing data. This applied to the stressors, social influence, and compliance items.
- Mean composite scores were calculated for the following variables:
  - Perceived Stress Scale (PSS-10): Four items were reverse scored (items 4, 5, 7, and 8) and a mean of 10 items was calculated.
  - Perceived Loneliness Scale (SLON-3): The scale was initially coded as an extension of the PSS-10 scale. For clarity, the items were renamed from perceived_stress_sca_11 through perceived_stress_sca_13 to Scale_SLON_1 through Scale_SLON_3 and averaged.
  - Perceived Support Scale (PSUP-3): Three items were averaged. Two versions of this composite were calculated with neutrals coded both as zero and as midpoints on the scale.
  - Vaccine Attitudes Scale: Two items were reverse scored (items 4 and 5) and six items were averaged; two versions of this composite were calculated with neutrals coded both as zero and as midpoints on the scale.
  - Resilience Scale: Three items were reverse scored (items 2, 4 and 6) and all six items were averaged.
  - Uncertainty: Five items were averaged.

## Technical Validation

Given that this is a large-scale survey distributed by numerous researchers all over the world, we had limited control over the total number of responses per country. In line with the first COVIDiSTRESS Global Survey project, in order to be considered for country-level analyses, a country needed at least 30 respondents for detecting both the effects of individual- and country-level predictors. In addition, a goal of 200 participants per country was set. The sample size considerations mirrored the first COVIDiSTRESS global survey project and were based on power simulation results for required sample size and group size to detect such effects with 80% statistical power^32^.

In order to be considered as a valid participant for the present analyses by country, a respondent must have reported their country of residence and submitted valid responses for the variables treated in each analysis. For inclusion in global analyses of a given variable, the participant only needed to submit valid responses for that variable. Participants were included in descriptive analyses for a given survey if they answered questions on that survey. If they selected a not applicable (NA) option for some items, these items were not included in their individual average. For reliability analyses, participants were only included if they answered all items on a given scale. Reliability testing was only performed for scales in which participants all received identical items. Items on the misperceptions and social norms scales were randomly selected from matched blocks of questions, so reliability testing was not conducted for these scales. Convergent validity will be further tested in follow-up pre-registered hypotheses tests of correlations between related variables.

For all composite scores used for this technical validation, neutral values were retained as the midpoint of the scale where they existed in the previous survey. After data cleaning and scale-wise exclusion of participants who did not complete any items on a given scale, additional scale composites were calculated in MS Excel/SPSS.

### Demographic characteristics

Data was collected from 137 countries, presented in Fig. 2 and coded according to the number of participants. A total of 28 countries had more than 200 participants, and 63 countries had more than 30 participants. After data cleaning, a total of 120 countries were represented with 25 countries each containing greater than 200 participants, 35 countries with over 100 participants, and 54 countries with more than 30 participants. The number of responses for both raw and cleaned data for countries with 30 or more participants are presented in Supplementary Table 1. Henceforth, all analyses are presented for the cleaned dataset only. Demographic information and response rate characteristics are presented for countries with more than 200 participants in Tables 1 through 5. Response rates are presented in Table 1. The following characteristics have been assessed by country: age and gender (Table 2), education (Table 3), marital status (Table 4), and COVID-19 history (Table 5). Additional demographic information can be obtained from the cleaned dataset.

We recognize that this dataset is not fully generalizable to all populations. It is important to note that this study was conducted as an expansion in the scope of the initial COVIDiSTRESS study (https://osf.io/z39us/)^1^ with the goal of reaching participants in underrepresented areas of the initial COVIDiSTRESS study: Russia, Africa, and Central Asia. There were over 200 respondents from Africa, 254 from Kyrgyzstan, and 2260 from Russia.

### Composite scoring and reliability testing of scales

**Fig. 3:**
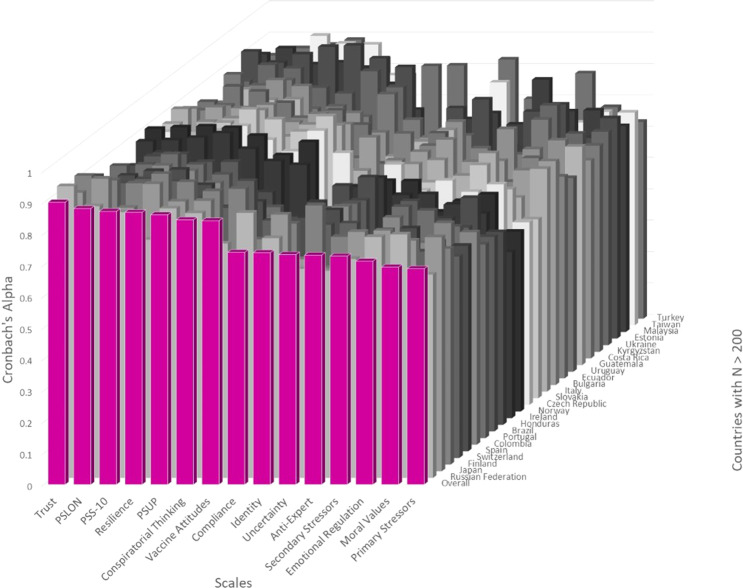
Reliability Values for Each Scale. Overall Cronbach’s alpha values for each survey are represented for the full dataset for each scale. Values for countries with N > 200 are represented on the z-axis to exhibit reliability across sub-samples.

Descriptive statistics and reliability testing for all scales combined across all countries are presented in Table 6. Cronbach’s alpha^33^ was calculated for each scale and determined to be unacceptable below 0.6, low but reliable from 0.6 to 0.7, respectable between 0.7 and 0.8, and good above 0.8, as is customary^34–36^ and recommended for maximal internal consistency of survey items without redundancy^37,38^. All scales have respectable internal reliability (Cronbach’s alpha > 0.7) for the full sample, except Moral Values (in which all subscales were combined, naturally reducing Cronbach’s alpha) and primary stressors, both of which neared 0.7 (0.694 and 0.689, respectively; Fig. 3). While a Cronbach’s alpha value below 0.7 would be expected for scales that are not unidimensional^35^, further factor analyses are recommended before using these two scales.

**Table 6:** Descriptive statistics and reliability testing for global data on all scales.

Scale	Composite Score Code	Min	Max	Mean	SD	#Items	N for Descriptives	N for Reliability	Cronbach’s Alpha
Identity	COM_Identity_4	1	7	4.83	1.12	4	15549	15549	0.740
Primary Stressors	COM_Primary_Stressors_4	0	4	1.88	1.02	4	15665	14842	0.689
Secondary Stressors	COM_Secondary_Stressors_14*	0	4	1.44	1.00	4	10516	9183	0.729
Perceived Support	COM_PSUP_3	1	7	5.05	1.44	3	15690	15690	0.861
Compliance	COM_Compliance	1	7	5.26	1.08	8	15530	12099	0.741
Social Norms	COM_SocialNorms	1	7	5.17	1.38	4 (of 16 total)	15344	**	**
Trust	COM_Trust	0	10	5.01	2.35	7	15068	15068	0.901
Misperceptions	COM_Misperceptions	1	7	2.27	1.21	3 (of 6 total)	13099	**	**
Conspiratorial Thinking	COM_Conspiratorial	1	7	3.65	1.52	4	12981	12981	0.845
Anti-Expert Sentiment	COM_AntiExpert	1	7	2.88	1.26	3	12939	12939	0.732
Moral Values	COM_MoralValues	1	7	5.06	0.76	11	12860	12860	0.694***
Emotional Regulation	COM_EmotionalRegulation	1	7	4.36	0.95	8	12898	12898	0.713
Loneliness	COM_PSLON_3	0	4	1.61	1.09	3	15661	15661	0.881
Perceived Stress	COM_PSS_10	0	4	1.87	0.69	10	15612	15612	0.872
Vaccine Attitudes	COM_VaccineAttitudes	1	7	4.99	1.33	6	15293	15293	0.842
Resilience	COM_Resilience	1	7	4.34	1.24	6	13248	13248	0.869
Intolerance of Uncertainty	COM_Uncertainty	1	5	2.77	0.81	5	13202	13202	0.734

*Note that while all items were included in descriptive analyses, alpha was calculated only with the first 4 items, as these were consistently shown to all participants. **Reliability not performed, as individual items were randomly presented to participants. ***Note that all subscales were combined for this analysis. Abbreviations: Composite Score Code = Composite score name used in all data files. Min = minimum score possible on a scale. Max = maximum score possible on per scale. Mean = global mean score on scale. SD = global standard deviation per scale. #Items = number of items on scale. N for Descriptives = number of subjects who answered questions on a scale. N for Reliability = number of participants who answered all questions with Likert/numerical responses on a scale. Cronbach’s Alpha = reliability testing for global data.

#### COM_Identity_4

The composite score for the Identity Scale was computed by averaging the four Identity items pertaining to identifying with family, local community, one’s country, and humanity. The basic descriptive statistics of the Identity Scale are summarized in Table 7. Specifically, 15,549 respondents completed this survey (98.8% of the participants). The composite scale score ranges from 1 to 7, with a mean value of 4.83 (SD = 1.12). The internal consistency of the scale, as measured by Cronbach’s alpha is 0.740 and ranges from 0.608 to 0.819.

**Table 7:** The basic descriptive statistics and reliability testing for the Identity Scale across countries with more than 200 participants.

Country	N Scale	Mean	SD	Min	Max	Alpha
Russian Federation	2193	4.87	1.10	1.0	7.0	0.765
Japan	2130	4.34	1.01	1.0	7.0	0.819
Finland	960	5.08	1.08	1.0	7.0	0.769
Switzerland	593	5.09	0.91	1.8	7.0	0.643
Spain	574	4.69	1.18	1.0	7.0	0.745
Colombia	548	4.69	1.21	1.0	7.0	0.746
Portugal	484	4.98	1.07	1.5	7.0	0.732
Brazil	447	4.68	1.12	1.0	7.0	0.673
Honduras	423	4.62	1.18	1.0	7.0	0.763
Ireland	401	5.31	1.02	1.0	7.0	0.738
Norway	376	5.29	1.07	1.0	7.0	0.718
Czech Republic	361	4.65	1.01	2.0	7.0	0.608
Slovakia	307	4.38	0.96	1.3	7.0	0.654
Italy	303	4.53	1.11	1.8	7.0	0.647
Bulgaria	284	4.89	1.18	1.0	7.0	0.717
Ecuador	286	5.05	1.21	1.0	7.0	0.781
Uruguay	286	5.10	0.94	1.5	7.0	0.634
Guatemala	287	5.06	1.16	1.0	7.0	0.713
Costa Rica	265	5.00	1.12	1.3	7.0	0.705
Kyrgyzstan	229	4.90	1.02	1.8	7.0	0.691
Ukraine	251	4.73	1.20	1.8	7.0	0.627
Estonia	243	4.69	0.90	1.3	7.0	0.735
Malaysia	225	5.11	0.93	1.0	7.0	0.679
Taiwan	221	4.80	0.97	1.3	7.0	0.650
Turkey	200	4.21	1.17	1.0	6.5	0.639

Abbreviations: N Scale = number of participants who completed the scale. Mean = scale mean. SD = scale standard deviation. Min = minimum value of the mean scale score for the sample. Max = maximum value of the average scale score for the sample. Alpha = Cronbach’s alpha.

#### COM_Primary_Stressors_4

The composite score for the Primary Stressors Scale was computed by averaging 4 items pertaining to primary stressors related to the participant or their family members catching COVID-19, as well as the ability to travel and meet with friends and family. The basic descriptive statistics of the Primary Stressors Scale are summarized in Table 8. Specifically, 15,549 respondents completed this survey with valid responses (98.8% of the participants). The composite scale score ranges from 0 to 4, with a mean value of 1.88 (SD = 1.02). Because respondents were presented with a not applicable option, only those who answered all questions with numerical responses (N = 14,842; 94.3% of participants) were included in the reliability analysis. The internal consistency of the scale, as measured by Cronbach’s alpha is 0.689 and ranges from 0.530 to 0.747.

**Table 8:** The basic descriptive statistics and reliability testing for the Primary Stressors Scale across countries with more than 200 participants.

Country	N Scale	N for Reliability	Mean	SD	Min	Max	Alpha
Russian Federation	2244	2046	1.61	0.96	0.0	4.0	0.649
Japan	2123	2039	1.86	1.04	0.0	4.0	0.747
Finland	960	915	1.44	0.94	0.0	4.0	0.689
Switzerland	592	563	1.58	0.91	0.0	4.0	0.643
Spain	573	549	2.03	0.87	0.0	4.0	0.656
Colombia	548	533	2.08	0.97	0.0	4.0	0.678
Portugal	483	471	2.16	0.90	0.0	4.0	0.618
Brazil	446	432	2.41	0.87	0.0	4.0	0.621
Honduras	427	391	2.04	0.89	0.0	4.0	0.615
Ireland	401	391	2.20	0.91	0.0	4.0	0.530
Norway	376	367	1.77	0.94	0.0	4.0	0.574
Czech Republic	365	335	1.46	0.95	0.0	4.0	0.583
Slovakia	313	292	1.92	0.83	0.0	4.0	0.570
Italy	307	294	1.82	1.01	0.0	4.0	0.711
Bulgaria	295	266	1.34	1.08	0.0	4.0	0.714
Ecuador	291	279	2.24	0.91	0.0	4.0	0.644
Uruguay	287	282	1.95	0.87	0.0	4.0	0.618
Guatemala	286	274	2.06	0.95	0.0	4.0	0.697
Costa Rica	270	261	2.21	0.87	0.3	4.0	0.632
Kyrgyzstan	250	231	1.89	0.98	0.0	4.0	0.658
Ukraine	252	232	1.46	0.93	0.0	4.0	0.681
Estonia	244	234	1.41	0.94	0.0	4.0	0.703
Malaysia	225	213	2.76	0.90	0.0	4.0	0.657
Taiwan	221	216	1.83	0.86	0.0	4.0	0.679
Turkey	199	189	2.08	0.97	0.0	4.0	0.628

Abbreviations: N Scale = number of participants who answered questions on the scale. N for reliability = number of subjects who selected a response on the Likert scale for every item (not “Does not apply to me). Mean = scale mean. SD = scale standard deviation. Min = minimum value of the mean scale score for the sample. Max = maximum value of the average scale score for the sample. Alpha = Cronbach’s alpha.

#### COM_Secondary_Stressors_14

The composite score for the Secondary Stressors Scale was computed by averaging 14 items pertaining to secondary stressors related to COVID-19’s impact on work, finances, education, relationships, and safety. Four of these items were presented to all participants, and the remainder were conditionally presented based on demographic information. The basic descriptive statistics of the Secondary Stressors Scale are summarized in Table 9. Specifically, 10,516 respondents completed this survey with valid responses (66.8% of the participants). The composite scale score ranges from 0 to 4, with a mean value of 1.44 (SD = 1.00). Because respondents were presented with a not applicable option, only those who answered all questions with numerical responses on the Likert scale (N = 9183; 58.3% of participants) were included in the reliability analysis. The internal consistency of the scale, as measured by Cronbach’s alpha is 0.729 and ranges from 0.540 to 0.805.

**Table 9:** The basic descriptive statistics and reliability testing for the Secondary Stressors Scale across countries with more than 200 participants.

Country	N Scale	N for Reliability	Mean	SD	Min	Max	Alpha
Russian Federation	1304	1112	1.60	1.01	0.0	4.0	0.699
Japan	1588	1463	1.53	1.02	0.0	4.0	0.764
Finland	730	622	0.84	0.75	0.0	4.0	0.662
Switzerland	389	338	0.81	0.76	0.0	4.0	0.692
Spain	400	379	1.47	0.91	0.0	4.0	0.706
Colombia	429	366	1.73	1.02	0.0	4.0	0.725
Portugal	221	182	1.42	1.00	0.0	4.0	0.738
Brazil	310	222	1.68	1.02	0.0	4.0	0.735
Honduras	169	151	2.01	0.95	0.0	4.0	0.666
Ireland	239	210	1.40	0.89	0.0	4.0	0.715
Norway	295	278	0.93	0.79	0.0	4.0	0.638
Czech Republic	268	216	1.20	0.90	0.0	4.0	0.716
Slovakia	186	164	1.77	0.93	0.0	4.0	0.630
Italy	202	171	1.42	0.94	0.0	4.0	0.689
Bulgaria	207	162	1.15	0.95	0.0	4.0	0.712
Ecuador	210	191	2.00	1.04	0.0	4.0	0.734
Uruguay	254	224	1.32	0.89	0.0	4.0	0.659
Guatemala	214	186	1.55	0.86	0.0	3.4	0.556
Costa Rica	218	200	1.75	1.02	0.0	4.0	0.732
Kyrgyzstan	162	129	1.88	0.99	0.0	4.0	0.630
Ukraine	213	189	1.10	0.79	0.0	3.3	0.540
Estonia	219	194	0.99	0.81	0.0	4.0	0.724
Malaysia	66	59	2.03	1.10	0.1	4.0	0.805
Taiwan	175	159	1.46	0.81	0.0	4.0	0.627
Turkey	43	40	2.00	0.93	0.4	4.0	0.658

Abbreviations: N Scale = number of participants who answered questions on the scale. N for reliability = number of subjects who selected a response on the Likert scale for every item (not “Does not apply to me). Mean = scale mean. SD = scale standard deviation. Min = minimum value of the mean scale score for the sample. Max = maximum value of the average scale score for the sample. Alpha = Cronbach’s alpha.

#### COM_PSUP_3

The composite score for the Perceived Support Scale (PSUP-3) was computed by averaging the three items regarding support networks. The basic descriptive statistics of the scale are summarized in Table 10. Specifically, 15,690 respondents completed this survey (99.7% of the participants). The composite scale score ranges from 1 to 7, with a mean value of 5.05 (SD = 1.44). The internal consistency of the scale, as measured by Cronbach’s alpha is 0.861 and ranges from 0.739 to 0.935.

**Table 10:** The basic descriptive statistics and reliability testing for the Perceived Support Scale (PSUP-3) across countries with more than 200 participants.

Country	N Scale	Mean	SD	Min	Max	Alpha
Russian Federation	2253	5.33	1.30	1.0	7.0	0.860
Japan	2129	3.96	1.37	1.0	7.0	0.861
Finland	962	5.24	1.50	1.0	7.0	0.902
Switzerland	592	5.58	1.11	1.0	7.0	0.829
Spain	575	5.50	1.33	1.0	7.0	0.850
Colombia	546	5.08	1.48	1.0	7.0	0.835
Portugal	484	5.35	1.25	1.0	7.0	0.841
Brazil	448	5.35	1.34	1.0	7.0	0.860
Honduras	427	4.71	1.38	1.0	7.0	0.778
Ireland	400	5.11	1.42	1.0	7.0	0.859
Norway	376	5.29	1.44	1.0	7.0	0.880
Czech Republic	364	5.32	1.33	1.0	7.0	0.846
Slovakia	312	5.16	1.36	1.0	7.0	0.890
Italy	308	4.81	1.48	1.0	7.0	0.814
Bulgaria	299	4.96	1.56	1.0	7.0	0.839
Ecuador	291	5.13	1.44	1.0	7.0	0.831
Uruguay	288	5.80	1.14	1.0	7.0	0.795
Guatemala	287	5.53	1.23	1.0	7.0	0.785
Costa Rica	270	5.43	1.29	1.0	7.0	0.739
Kyrgyzstan	251	5.14	1.22	1.0	7.0	0.783
Ukraine	252	5.27	1.43	1.3	7.0	0.803
Estonia	246	5.01	1.40	1.0	7.0	0.935
Malaysia	225	4.87	1.35	1.0	7.0	0.843
Taiwan	221	5.15	1.19	1.0	7.0	0.896
Turkey	199	4.77	1.53	1.0	7.0	0.828

Abbreviations: N Scale = number of participants who completed the scale. Mean = scale mean. SD = scale standard deviation. Min = minimum value of the mean scale score for the sample. Max = maximum value of the average scale score for the sample. Alpha = Cronbach’s alpha.

#### COM_Compliance

The composite score for the Compliance Scale was computed by averaging 8 items pertaining to compliance with guidelines to reduce the spread of COVID-19. The basic descriptive statistics of the Compliance Scale are summarized in Table 11. Specifically, 15,530 respondents completed this survey with valid responses (98.7% of the participants). The composite scale score ranges from 1 to 7, with a mean value of 5.26 (SD = 1.08). Because respondents were presented with a not applicable option, only those who answered all questions with numerical responses (N = 12,099; 76.9%) were included in the reliability analysis. The internal consistency of the scale, as measured by Cronbach’s alpha is 0.741 and ranges from 0.355 to 0.846.

**Table 11:** The basic descriptive statistics and reliability testing for the Compliance Scale across countries with more than 200 participants.

Country	N Scale	N for Reliability	Mean	SD	Min	Max	Alpha
Russian Federation	2235	2163	4.77	1.24	1.0	7.0	0.846
Japan	2125	1483	5.04	0.86	1.0	7.0	0.651
Finland	952	654	5.12	1.05	1.0	7.0	0.719
Switzerland	586	359	5.16	1.07	1.0	7.0	0.758
Spain	560	451	5.66	0.92	1.0	7.0	0.661
Colombia	540	434	5.86	0.86	1.0	7.0	0.668
Portugal	480	264	5.84	0.65	2.6	7.0	0.443
Brazil	445	291	5.97	0.62	3.1	7.0	0.424
Honduras	423	368	5.82	0.81	2.0	7.0	0.711
Ireland	393	243	5.49	0.86	1.8	7.0	0.623
Norway	372	271	5.17	0.99	2.3	7.0	0.646
Czech Republic	359	265	4.70	1.27	1.0	7.0	0.804
Slovakia	308	228	5.21	0.94	1.0	7.0	0.679
Italy	304	211	5.38	1.02	1.6	7.0	0.705
Bulgaria	292	256	4.32	1.36	1.0	7.0	0.790
Ecuador	286	223	5.83	0.80	1.7	7.0	0.580
Uruguay	283	225	5.56	0.92	1.6	7.0	0.664
Guatemala	283	241	5.71	0.90	1.0	7.0	0.666
Costa Rica	268	213	5.78	0.79	2.8	7.0	0.610
Kyrgyzstan	245	240	5.07	0.98	1.9	7.0	0.740
Ukraine	250	210	4.62	0.96	1.5	6.9	0.587
Estonia	240	158	4.94	0.92	1.0	7.0	0.581
Malaysia	224	183	6.11	0.51	3.3	7.0	0.355
Taiwan	220	176	5.73	0.58	4.3	7.0	0.455
Turkey	196	167	5.86	1.06	1.0	7.0	0.808

Abbreviations: N Scale = number of participants who answered questions on the scale. N for reliability = number of subjects who selected a response on the Likert scale for every item (not “Does not apply to me). Mean = scale mean. SD = scale standard deviation. Min = minimum value of the mean scale score for the sample. Max = maximum value of the average scale score for the sample. Alpha = Cronbach’s alpha.

#### COM_SocialNorms

The Social Influence Norms Scale contained 16 items across two corresponding blocks; 2 items from each block were randomly presented to each participant. To compute the composite score, two items were reverse scored (item 7 from each block) and the 4 items for each participant were averaged. A total of 15,344 respondents completed the survey with valid responses (97.5% of participants). The composite scale score ranges from 1 to 7, with a mean value of 5.17 (SD = 1.38). Descriptive statistics for this scale are summarized in Table 12.

**Table 12:** The basic descriptive statistics for the Social Norms Scale across countries with more than 200 participants.

Country	N Scale	Mean	SD	Min	Max
Russian Federation	2224	4.59	1.43	1.0	7.0
Japan	2113	5.40	1.11	1.0	7.0
Finland	938	5.00	1.31	1.0	7.0
Switzerland	585	4.90	1.28	1.0	7.0
Spain	554	5.40	1.31	1.0	7.0
Colombia	529	5.81	1.17	1.0	7.0
Portugal	474	5.63	1.14	1.0	7.0
Brazil	442	5.51	1.33	1.0	7.0
Honduras	423	5.98	1.14	2.0	7.0
Ireland	380	5.16	1.23	1.3	7.0
Norway	366	5.02	1.34	1.0	7.0
Czech Republic	351	4.29	1.54	1.0	7.0
Slovakia	298	5.04	1.35	1.0	7.0
Italy	301	5.18	1.37	1.0	7.0
Bulgaria	290	4.12	1.78	1.0	7.0
Ecuador	285	5.94	1.10	1.5	7.0
Uruguay	279	5.60	1.27	1.0	7.0
Guatemala	279	5.65	1.19	1.0	7.0
Costa Rica	265	5.72	1.16	1.0	7.0
Kyrgyzstan	239	4.86	1.37	1.0	7.0
Ukraine	248	4.64	1.21	1.8	7.0
Estonia	237	5.08	1.31	1.0	7.0
Malaysia	221	6.04	0.98	1.0	7.0
Taiwan	220	5.71	1.02	2.5	7.0
Turkey	189	5.04	1.19	1.5	7.0

Abbreviations: N Scale = number of participants who answered questions on the scale. Mean = scale mean. SD = scale standard deviation. Min = minimum value of the mean scale score for the sample. Max = maximum value of the average scale score for the sample.

#### COM_Trust

The composite score for the Trust Scale was computed by averaging seven items pertaining to trust in national government, health and security, scientists, and the World Health Organization. The basic descriptive statistics of the scale are summarized in Table 13. A total of 15,068 respondents completed this survey (95.7% of the participants). The composite scale score ranges from 0 to 10, with a mean value of 5.01 (SD = 2.35). The internal consistency of the scale, as measured by Cronbach’s alpha is 0.901 and ranges from 0.589 to 0.931.

**Table 13:** The basic descriptive statistics and reliability testing for the Trust Scale across countries with more than 200 participants.

Country	N Scale	Mean	SD	Min	Max	Alpha
Russian Federation	2197	4.26	2.28	0.0	10.0	0.931
Japan	2099	4.39	1.87	0.0	10.0	0.889
Finland	923	7.31	1.97	0.0	10.0	0.922
Switzerland	578	7.39	1.71	0.6	10.0	0.900
Spain	547	5.90	1.85	0.0	10.0	0.847
Colombia	511	4.19	1.80	0.0	10.0	0.824
Portugal	458	6.46	1.69	0.0	10.0	0.869
Brazil	438	4.67	1.23	1.4	8.9	0.589
Honduras	402	2.65	1.66	0.0	9.4	0.827
Ireland	369	5.96	1.95	0.3	10.0	0.883
Norway	360	7.05	2.00	1.3	10.0	0.902
Czech Republic	344	4.58	2.02	0.0	8.9	0.871
Slovakia	298	4.42	1.93	0.0	9.3	0.876
Italy	294	5.23	2.25	0.0	9.6	0.902
Bulgaria	285	2.73	1.99	0.0	8.4	0.882
Ecuador	272	4.27	1.79	0.0	9.0	0.855
Uruguay	276	6.16	1.94	0.6	10.0	0.861
Guatemala	272	2.72	1.43	1.0	7.1	0.738
Costa Rica	260	5.93	1.69	1.3	9.3	0.815
Kyrgyzstan	231	2.70	1.83	0.0	8.7	0.885
Ukraine	247	4.19	1.69	0.0	10.0	0.842
Estonia	235	6.66	1.98	0.0	10.0	0.916
Malaysia	212	5.34	2.06	0.0	9.3	0.886
Taiwan	219	6.93	1.18	0.7	10.0	0.774
Turkey	180	4.63	1.97	0.0	9.4	0.840

Abbreviations: N Scale = number of participants who completed the scale. Mean = scale mean. SD = scale standard deviation. Min = minimum value of the mean scale score for the sample. Max = maximum value of the average scale score for the sample. Alpha = Cronbach’s alpha.

#### COM_Misperceptions

Six items regarding misperceptions about COVID-19 were divided into 3 blocks, with 1 item randomly presented to participants from each block. Two items were reversed scored (items 1 and 2) and the three presented items were averaged for each participant. The composite score for the Misperceptions Scale was computed by averaging three (of six total) items. The basic descriptive statistics of the scale are summarized in Table 14. A total of 13,099 respondents completed this survey (83.2% of the participants). The composite scale score ranges from 1 to 7, with a mean value of 2.27 (SD = 1.21).

**Table 14:** The basic descriptive statistics for the Misperceptions about COVID-19 Scale across countries with more than 200 participants.

Country	N Scale	Mean	SD	Min	Max
Russian Federation	1794	3.03	1.21	1.0	7.0
Japan	2016	2.51	1.09	1.0	7.0
Finland	887	1.85	1.07	1.0	7.0
Switzerland	527	1.76	0.91	1.0	7.0
Spain	485	1.79	0.97	1.0	7.0
Colombia	446	1.87	1.08	1.0	6.7
Portugal	387	1.71	0.85	1.0	5.3
Brazil	392	1.41	0.68	1.0	6.0
Honduras	307	2.60	1.18	1.0	6.3
Ireland	310	1.70	0.90	1.0	5.3
Norway	328	1.79	0.89	1.0	6.3
Czech Republic	298	2.16	1.17	1.0	7.0
Slovakia	269	2.25	1.18	1.0	6.0
Italy	271	2.11	1.20	1.0	6.7
Bulgaria	262	3.25	1.53	1.0	7.0
Ecuador	210	2.28	1.20	1.0	6.3
Uruguay	218	2.17	1.16	1.0	6.3
Guatemala	223	2.16	1.16	1.0	7.0
Costa Rica	218	2.06	1.12	1.0	6.7
Kyrgyzstan	191	3.20	1.31	1.0	7.0
Ukraine	217	1.83	0.97	1.0	6.3
Estonia	206	1.85	0.90	1.0	6.0
Malaysia	168	1.98	1.05	1.0	5.3
Taiwan	198	2.24	1.00	1.0	5.7
Turkey	146	2.27	1.02	1.0	5.3

Abbreviations: N Scale = number of participants who answered questions on the scale. Mean = scale mean. SD = scale standard deviation. Min = minimum value of the mean scale score for the sample. Max = maximum value of the average scale score for the sample.

#### COM_Conspiratorial

The composite score for the Conspiratorial Thinking Scale was computed by averaging four items about conspiratorial thinking. The basic descriptive statistics of the scale are summarized in Table 15. A total of 12,981 respondents completed this survey (82.5% of the participants). The composite scale score ranges from 1 to 7, with a mean value of 3.65 (SD = 1.52). The internal consistency of the scale, as measured by Cronbach’s alpha is 0.845 and ranges from 0.669 to 0.894.

**Table 15:** The basic descriptive statistics and reliability testing for the Conspiratorial Thinking Scale across countries with more than 200 participants.

Country	N Scale	Mean	SD	Min	Max	Alpha
Russian Federation	1782	4.21	1.39	1.0	7.0	0.845
Japan	1990	4.09	1.30	1.0	7.0	0.863
Finland	876	2.40	1.41	1.0	7.0	0.873
Switzerland	525	2.57	1.17	1.0	7.0	0.798
Spain	480	3.59	1.31	1.0	7.0	0.779
Colombia	448	3.95	1.37	1.0	7.0	0.764
Portugal	384	2.99	1.26	1.0	7.0	0.782
Brazil	390	3.58	1.14	1.0	7.0	0.669
Honduras	301	4.68	1.28	1.0	7.0	0.742
Ireland	308	2.92	1.30	1.0	7.0	0.820
Norway	327	2.46	1.25	1.0	7.0	0.818
Czech Republic	297	3.46	1.38	1.0	7.0	0.809
Slovakia	266	3.41	1.40	1.0	7.0	0.851
Italy	268	3.37	1.48	1.0	7.0	0.852
Bulgaria	258	4.43	1.53	1.0	7.0	0.865
Ecuador	210	3.92	1.41	1.0	7.0	0.797
Uruguay	216	3.34	1.43	1.0	7.0	0.834
Guatemala	221	4.40	1.26	1.0	7.0	0.723
Costa Rica	218	4.39	1.41	1.0	7.0	0.783
Kyrgyzstan	189	4.68	1.15	1.0	7.0	0.750
Ukraine	215	2.64	1.56	1.0	7.0	0.874
Estonia	205	2.29	1.26	1.0	7.0	0.894
Malaysia	168	4.12	1.28	1.0	7.0	0.791
Taiwan	196	3.25	1.34	1.0	6.5	0.798
Turkey	145	4.45	1.45	1.0	7.0	0.776

Abbreviations: N Scale = number of participants who completed the scale. Mean = scale mean. SD = scale standard deviation. Min = minimum value of the mean scale score for the sample. Max = maximum value of the average scale score for the sample. Alpha = Cronbach’s alpha.

#### COM_AntiExpert

The composite score for the Anti-Expert Sentiment Scale was computed by averaging three items. The basic descriptive statistics of the scale are summarized in Table 16. A total of 12,939 respondents completed this survey (82.2% of the participants). The composite scale score ranges from 1 to 7, with a mean value of 2.88 (SD = 1.26). The internal consistency of the scale, as measured by Cronbach’s alpha is 0.732 and ranges from 0.412 to 0.783.

**Table 16:** The basic descriptive statistics and reliability testing for the Anti-Expert Scale across countries with more than 200 participants.

Country	N Scale	Mean	SD	Min	Max	Alpha
Russian Federation	1768	3.86	1.13	1.0	7.0	0.661
Japan	1988	3.20	0.99	1.0	7.0	0.648
Finland	882	2.17	1.09	1.0	7.0	0.727
Switzerland	524	2.44	1.10	1.0	7.0	0.749
Spain	483	2.38	1.11	1.0	6.7	0.626
Colombia	439	2.30	1.06	1.0	7.0	0.617
Portugal	385	2.84	1.05	1.0	6.3	0.642
Brazil	391	2.10	0.96	1.0	6.0	0.612
Honduras	290	3.08	1.17	1.0	7.0	0.659
Ireland	305	2.19	1.06	1.0	6.7	0.712
Norway	326	1.99	0.99	1.0	7.0	0.735
Czech Republic	296	2.77	1.16	1.0	7.0	0.684
Slovakia	266	2.47	1.04	1.0	5.7	0.689
Italy	264	2.94	1.33	1.0	6.7	0.739
Bulgaria	256	3.62	1.46	1.0	7.0	0.783
Ecuador	208	2.51	1.08	1.0	7.0	0.623
Uruguay	215	2.21	0.88	1.0	7.0	0.589
Guatemala	219	2.70	1.06	1.0	7.0	0.608
Costa Rica	215	2.29	1.03	1.0	5.7	0.605
Kyrgyzstan	192	4.11	1.03	1.3	7.0	0.540
Ukraine	215	2.74	1.04	1.0	6.0	0.412
Estonia	206	2.32	1.00	1.0	5.3	0.614
Malaysia	167	2.66	1.05	1.0	6.3	0.662
Taiwan	198	3.53	1.05	1.0	6.0	0.539
Turkey	148	3.22	1.18	1.0	7.0	0.701

Abbreviations: N Scale = number of participants who completed the scale. Mean = scale mean. SD = scale standard deviation. Min = minimum value of the mean scale score for the sample. Max = maximum value of the average scale score for the sample. Alpha = Cronbach’s alpha.

#### COM_MoralValues

The composite score for the Moral Values Scale was computed by averaging 11 items. The basic descriptive statistics of the scale are summarized in Table 17. A total of 12,860 respondents completed this survey (81.7% of the participants). The composite scale score ranges from 1 to 7, with a mean value of 5.06 (SD = 0.76). The internal consistency of the scale, as measured by Cronbach’s alpha is 0.694, and ranges from 0.601 to 0.777. However, it should be noted that all subscales of were combined for this analysis. Separating by subscale according to the original scale^28,29^ is recommended for future analyses using this data.

**Table 17:** The basic descriptive statistics and reliability testing for the Moral Values Scale across countries with more than 200 participants.

Country	N Scale	Mean	SD	Min	Max	Alpha
Russian Federation	1774	5.22	0.78	1.0	7.0	0.777
Japan	2014	4.85	0.67	1.0	7.0	0.736
Finland	863	5.03	0.66	3.1	7.0	0.619
Switzerland	514	4.98	0.65	3.0	7.0	0.659
Spain	469	4.84	0.72	1.0	6.7	0.669
Colombia	439	5.14	0.77	1.0	7.0	0.707
Portugal	380	5.09	0.67	2.5	6.7	0.675
Brazil	384	4.78	0.65	2.6	6.5	0.651
Honduras	296	5.44	0.78	1.5	7.0	0.724
Ireland	301	5.08	0.69	3.1	6.8	0.653
Norway	320	4.96	0.70	2.7	6.8	0.693
Czech Republic	291	4.94	0.73	1.5	6.7	0.704
Slovakia	263	5.15	0.61	2.8	6.8	0.667
Italy	261	5.07	0.65	3.0	6.8	0.619
Bulgaria	259	5.48	0.69	3.2	7.0	0.685
Ecuador	205	5.32	0.78	1.7	7.0	0.703
Uruguay	214	5.20	0.64	3.6	7.0	0.601
Guatemala	217	5.23	0.71	3.5	6.7	0.642
Costa Rica	215	5.16	0.76	3.0	7.0	0.696
Kyrgyzstan	184	5.27	0.61	3.0	6.7	0.610
Ukraine	209	4.68	0.80	1.5	6.8	0.675
Estonia	204	5.02	0.60	3.2	6.6	0.640
Malaysia	168	5.46	0.73	3.6	7.0	0.707
Taiwan	196	4.48	0.71	2.4	6.7	0.681
Turkey	141	5.13	0.74	2.5	6.6	0.675

Abbreviations: N Scale = number of participants who completed the scale. Mean = scale mean. SD = scale standard deviation. Min = minimum value of the mean scale score for the sample. Max = maximum value of the average scale score for the sample. Alpha = Cronbach’s alpha.

#### COM_EmotionalRegulation

The composite score for the Emotion Regulation Scale was computed by averaging eight items. The basic descriptive statistics of the scale are summarized in Table 18. A total of 12,898 respondents completed this survey (81.9% of the participants). The composite scale score ranges from 1 to 7, with a mean value of 4.36 (SD = 0.95). The internal consistency of the scale, as measured by Cronbach’s alpha is 0.713 and ranges from 0.541 to 0.873.

**Table 18:** The basic descriptive statistics and reliability testing for the Emotion Regulation Scale across countries with more than 200 participants.

Country	N Scale	Mean	SD	Min	Max	Alpha
Russian Federation	1764	4.44	1.09	1.0	7.0	0.769
Japan	2013	4.45	0.78	1.0	7.0	0.768
Finland	877	4.18	0.77	1.5	6.5	0.657
Switzerland	522	3.96	0.71	1.5	6.1	0.541
Spain	472	4.22	0.98	1.0	6.9	0.712
Colombia	434	4.38	0.97	1.0	7.0	0.644
Portugal	381	4.29	0.96	1.4	6.8	0.726
Brazil	388	4.19	0.89	1.4	6.8	0.645
Honduras	305	4.80	0.94	2.4	7.0	0.659
Ireland	301	4.29	0.95	1.9	7.0	0.682
Norway	317	3.97	0.87	1.3	7.0	0.672
Czech Republic	296	4.12	0.87	1.5	7.0	0.680
Slovakia	265	4.23	0.94	1.4	6.6	0.744
Italy	266	4.17	1.04	1.5	7.0	0.761
Bulgaria	258	4.44	0.99	1.0	7.0	0.715
Ecuador	206	4.46	0.91	1.9	7.0	0.627
Uruguay	213	4.12	0.83	1.6	6.3	0.579
Guatemala	215	4.38	0.86	1.8	6.6	0.575
Costa Rica	216	4.37	0.98	1.3	6.6	0.668
Kyrgyzstan	188	4.75	1.04	1.5	7.0	0.741
Ukraine	209	4.25	1.00	1.0	6.9	0.765
Estonia	205	4.36	0.89	1.4	6.6	0.747
Malaysia	167	4.76	0.96	2.1	7.0	0.704
Taiwan	197	4.60	0.81	2.4	6.6	0.647
Turkey	145	4.50	1.09	2.1	7.0	0.783

Abbreviations: N Scale = number of participants who completed the scale. Mean = scale mean. SD = scale standard deviation. Min = minimum value of the mean scale score for the sample. Max = maximum value of the average scale score for the sample. Alpha = Cronbach’s alpha.

#### COM_PSLON_3

The composite score for the Loneliness Scale (SLON-3) was computed by averaging three items of the extended PSS-10 Scale. The basic descriptive statistics of the scale are summarized in Table 19. A total of 15,661 respondents completed this survey (99.5% of the participants). The composite scale score ranges from 0 to 4, with a mean value of 1.61 (SD = 1.09). The internal consistency of the scale, as measured by Cronbach’s alpha is 0.881 and ranges from 0.836 to 0.934.

**Table 19:** The basic descriptive statistics and reliability testing for the Loneliness Scale (SLON-3) across countries with more than 200 participants.

Country	N Scale	Mean	SD	Min	Max	Alpha
Russian Federation	2249	1.47	1.05	0.0	4.0	0.867
Japan	2129	1.53	1.04	0.0	4.0	0.934
Finland	961	1.54	1.09	0.0	4.0	0.907
Switzerland	592	1.20	0.97	0.0	4.0	0.877
Spain	574	1.49	1.04	0.0	4.0	0.880
Colombia	548	1.59	1.08	0.0	4.0	0.852
Portugal	484	1.63	1.10	0.0	4.0	0.873
Brazil	448	1.78	1.13	0.0	4.0	0.878
Honduras	423	1.74	1.07	0.0	4.0	0.869
Ireland	401	2.07	1.17	0.0	4.0	0.887
Norway	376	1.79	1.18	0.0	4.0	0.907
Czech Republic	364	1.89	1.06	0.0	4.0	0.863
Slovakia	312	2.00	1.00	0.0	4.0	0.836
Italy	309	1.63	1.01	0.0	4.0	0.861
Bulgaria	296	1.41	1.12	0.0	4.0	0.886
Ecuador	291	1.62	1.06	0.0	4.0	0.871
Uruguay	288	1.42	1.08	0.0	4.0	0.910
Guatemala	287	1.52	1.09	0.0	4.0	0.880
Costa Rica	269	1.68	1.06	0.0	4.0	0.845
Kyrgyzstan	244	1.34	0.98	0.0	4.0	0.850
Ukraine	252	1.91	0.07	0.0	4.0	0.894
Estonia	245	1.39	1.07	0.0	4.0	0.880
Malaysia	225	2.18	1.19	0.0	4.0	0.905
Taiwan	221	1.60	0.93	0.0	4.0	0.856
Turkey	200	2.06	1.15	0.0	4.0	0.863

Abbreviations: N Scale = number of participants who completed the scale. Mean = scale mean. SD = scale standard deviation. Min = minimum value of the mean scale score for the sample. Max = maximum value of the average scale score for the sample. Alpha = Cronbach’s alpha.

#### COM_PSS_10

The composite score for the Perceived Stress Scale (PSS-10) was computed by averaging 10 items about stress in the past month, four of which were reverse scored (items 4, 5, 7, and 8). The basic descriptive statistics of the scale are summarized in Table 20. A total of 15,612 respondents completed this survey (99.2% of the participants). The composite scale score ranges from 0 to 4, with a mean value of 1.87 (SD = 0.69). The internal consistency of the scale, as measured by Cronbach’s alpha is 0.872 and ranges from 0.810 to 0.924.

**Table 20:** The basic descriptive statistics and reliability testing for the Perceived Stress Scale (PSS-10) across countries with more than 200 participants.

Country	N Scale	Mean	SD	Min	Max	Alpha
Russian Federation	2233	1.83	0.60	0.0	3.9	0.848
Japan	2123	1.85	0.61	0.0	4.0	0.841
Finland	959	1.44	0.72	0.0	3.6	0.898
Switzerland	591	1.42	0.65	0.0	3.5	0.890
Spain	575	1.91	0.69	0.0	3.7	0.895
Colombia	547	1.91	0.69	0.1	1.0	0.891
Portugal	484	2.05	0.75	0.0	3.9	0.906
Brazil	448	2.11	0.73	0.1	3.7	0.885
Honduras	422	1.96	0.57	0.0	4.0	0.810
Ireland	400	2.11	0.71	0.0	3.9	0.895
Norway	376	1.71	0.75	0.0	3.8	0.910
Czech Republic	362	1.96	0.68	0.2	4.0	0.875
Slovakia	311	2.01	0.65	0.3	3.9	0.879
Italy	305	1.90	0.68	0.1	3.4	0.872
Bulgaria	292	1.85	0.75	0.0	4.0	0.883
Ecuador	289	1.93	0.62	0.0	3.5	0.859
Uruguay	286	1.72	0.66	0.3	3.4	0.894
Guatemala	287	1.93	0.61	0.5	3.3	0.846
Costa Rica	270	1.92	0.69	0.0	3.7	0.888
Kyrgyzstan	243	1.78	0.62	0.3	3.3	0.867
Ukraine	252	1.90	0.68	0.0	3.6	0.883
Estonia	245	1.64	0.70	0.0	3.8	0.907
Malaysia	224	2.21	0.63	0.9	4.0	0.854
Taiwan	221	1.86	0.67	0.3	3.8	0.924
Turkey	199	2.46	0.64	0.8	4.0	0.874

Abbreviations: N Scale = number of participants who completed the scale. Mean = scale mean. SD = scale standard deviation. Min = minimum value of the mean scale score for the sample. Max = maximum value of the average scale score for the sample. Alpha = Cronbach’s alpha.

#### COM_VaccineAttitudes

The composite score for the Vaccine Attitudes Scale was computed by averaging six items about vaccine hesitancy, after reverse scoring two items (items 4 and 5. The basic descriptive statistics of the scale are summarized in Table 21. A total of 15,293 respondents completed this survey (97.2% of the participants). The composite scale score ranges from 1 to 7, with a mean value of 4.99 (SD = 1.33). The internal consistency of the scale, as measured by Cronbach’s alpha is 0.842 and ranges from 0.256 to 0.900.

**Table 21:** The basic descriptive statistics and reliability testing for the Vaccine Attitude Scale across countries with more than 200 participants.

Country	N Scale	Mean	SD	Min	Max	Alpha
Russian Federation	2228	3.82	1.15	1.0	7.0	0.748
Japan	2123	4.45	0.85	1.0	7.0	0.691
Finland	939	5.59	1.30	1.0	7.0	0.880
Switzerland	586	5.18	1.34	1.0	7.0	0.884
Spain	550	5.68	1.06	1.0	7.0	0.797
Colombia	520	5.83	0.92	1.5	7.0	0.726
Portugal	468	5.80	0.89	1.0	7.0	0.737
Brazil	441	6.40	0.63	2.8	7.0	0.568
Honduras	421	5.09	0.90	1.7	7.0	0.665
Ireland	374	5.43	1.14	1.0	7.0	0.808
Norway	364	5.43	1.32	1.0	7.0	0.860
Czech Republic	347	4.73	1.56	1.0	7.0	0.875
Slovakia	301	4.97	1.45	1.0	7.0	0.900
Italy	300	5.14	1.40	1.0	7.0	0.849
Bulgaria	290	4.13	1.53	1.0	7.0	0.832
Ecuador	280	5.48	0.98	2.0	7.0	0.703
Uruguay	280	5.44	1.09	1.0	7.0	0.788
Guatemala	278	5.31	1.08	1.0	7.0	0.727
Costa Rica	263	5.69	0.96	1.0	7.0	0.721
Kyrgyzstan	240	3.88	1.28	1.0	7.0	0.822
Ukraine	246	5.46	1.20	1.0	7.0	0.823
Estonia	236	5.39	1.20	1.0	7.0	0.866
Malaysia	216	5.53	0.83	2.0	7.0	0.709
Taiwan	220	5.26	0.60	3.7	6.7	0.256
Turkey	186	5.42	1.14	1.0	7.0	0.806

Abbreviations: N Scale = number of participants who completed the scale. Mean = scale mean. SD = scale standard deviation. Min = minimum value of the mean scale score for the sample. Max = maximum value of the average scale score for the sample. Alpha = Cronbach’s alpha.

#### COM_Resilience

The composite score for the Brief Resilience Scale was computed by averaging six items about resilience, three of which were reverse scored (items 2, 4 and 6). The basic descriptive statistics of the scale are summarized in Table 22. A total of 13,248 respondents completed this survey (84.2% of the participants). The composite scale score ranges from 1 to 7, with a mean value of 4.34 (SD = 1.24). The internal consistency of the scale, as measured by Cronbach’s alpha is 0.869 and ranges from 0.760 to 0.931.

**Table 22:** The basic descriptive statistics and reliability testing for the Brief Resilience Scale across countries with more than 200 participants.

Country	N Scale	Mean	SD	Min	Max	Alpha
Russian Federation	1802	4.41	1.05	1.0	7.0	0.760
Japan	2024	3.77	1.22	1.0	7.0	0.917
Finland	901	4.65	1.37	1.0	7.0	0.928
Switzerland	526	4.88	1.11	1.3	7.0	0.883
Spain	492	4.47	1.28	1.0	7.0	0.899
Colombia	454	4.51	1.33	1.0	7.0	0.874
Portugal	394	4.28	1.21	1.0	7.0	0.873
Brazil	397	4.17	1.24	1.0	7.0	0.860
Honduras	311	4.38	1.06	1.0	7.0	0.787
Ireland	316	4.38	1.33	1.0	7.0	0.909
Norway	329	4.73	1.29	1.0	7.0	0.905
Czech Republic	299	4.12	1.23	1.0	7.0	0.890
Slovakia	271	3.94	1.27	1.2	7.0	0.922
Italy	273	4.34	1.37	1.0	7.0	0.898
Bulgaria	260	4.65	1.29	1.0	7.0	0.894
Ecuador	213	4.47	1.06	1.2	7.0	0.788
Uruguay	225	4.62	1.17	1.5	7.0	0.858
Guatemala	226	4.66	1.14	2.0	7.0	0.848
Costa Rica	223	4.58	1.26	1.0	7.0	0.869
Kyrgyzstan	190	4.44	1.08	2.0	7.0	0.775
Ukraine	223	3.95	1.21	1.0	6.2	0.821
Estonia	210	4.34	1.13	1.5	7.0	0.931
Malaysia	173	4.20	0.09	1.5	7.0	0.860
Taiwan	200	4.50	1.10	1.0	6.5	0.899
Turkey	152	4.18	1.28	1.2	7.0	0.890

Abbreviations: N Scale = number of participants who completed the scale. Mean = scale mean. SD = scale standard deviation. Min = minimum value of the mean scale score for the sample. Max = maximum value of the average scale score for the sample. Alpha = Cronbach’s alpha.

#### COM_Uncertainty

The composite score for the Intolerance of Uncertainty Scale (IUS-5) was computed by averaging five items about desire for certainty. The basic descriptive statistics of the scale are summarized in Table 23. A total of 13,202 respondents completed this survey (83.9% of the participants). The composite scale score ranges from 1 to 5, with a mean value of 2.77 (SD = 0.81). The internal consistency of the scale, as measured by Cronbach’s alpha is 0.734 and ranges from 0.558 to 0.827.

**Table 23:** The basic descriptive statistics and reliability testing for the Intolerance of Uncertainty Scale (IUS-5) across countries with more than 200 participants.

Country	N Scale	Mean	SD	Min	Max	Alpha
Russian Federation	1799	2.96	0.74	1.0	5.0	0.667
Japan	2021	2.92	0.72	1.0	5.0	0.677
Finland	896	2.49	0.86	1.0	5.0	0.827
Switzerland	526	2.55	0.69	1.0	4.8	0.744
Spain	490	2.60	0.84	1.0	5.0	0.770
Colombia	453	2.53	0.82	1.0	5.0	0.760
Portugal	391	2.80	0.85	1.0	5.0	0.748
Brazil	396	2.98	0.81	1.2	5.0	0.737
Honduras	307	2.64	0.89	1.0	5.0	0.790
Ireland	311	2.80	0.84	1.0	5.0	0.767
Norway	329	2.45	0.77	1.0	4.8	0.736
Czech Republic	300	2.77	0.89	1.0	5.0	0.767
Slovakia	270	2.79	0.71	1.0	4.8	0.664
Italy	271	2.63	0.81	1.0	4.8	0.726
Bulgaria	261	2.79	0.80	1.0	5.0	0.744
Ecuador	213	2.56	0.80	1.0	5.0	0.742
Uruguay	223	2.33	0.75	1.0	4.2	0.761
Guatemala	227	2.66	0.81	1.0	5.0	0.750
Costa Rica	224	2.53	0.81	1.0	5.0	0.755
Kyrgyzstan	191	2.94	0.76	1.2	5.0	0.690
Ukraine	221	2.87	0.72	1.0	4.8	0.558
Estonia	209	2.64	0.81	1.0	4.8	0.763
Malaysia	171	3.19	0.77	1.2	5.0	0.698
Taiwan	199	2.82	0.80	1.2	5.0	0.775
Turkey	152	3.42	0.95	1.2	5.0	0.827

Abbreviations: N Scale = number of participants who completed the scale. Mean = scale mean. SD = scale standard deviation. Min = minimum value of the mean scale score for the sample. Max = maximum value of the average scale score for the sample. Alpha = Cronbach’s alpha.

## Usage Notes

We recommend that any interested researchers use the cleaned version of data (available at https://osf.io/36tsd/ under the CC-By Attribution 4.0 International license). Before using the dataset, we recommend consulting the R codebook and accompanying measured variables list. Variables can be used individually or with the calculated composites. To identify individuals from a specific country, the variable, ‘residing_country,’ should be used.

Composite scores were obtained for some variables using means, but it should be noted that for some validated scales used in this survey, other methods of computation were indicated in the original publications. Therefore, the raw dataset is available so that these scales can be recalculated as needed. In the raw data, a value of 99 means that the item does not apply for that individual; this distinction between not applicable and missing data has been preserved in the cleaned dataset in columns containing the extension “NAppl.” Neutral values were also added to some scales. Composite scores were calculated by coding neutral responses both as midpoint values (as presented in the survey) and as zero value responses. This was for the convenience of researchers using the data, but it should be noted that all technical validations were performed on data with neutrals coded as midpoints–as they were presented to participants.

Due to snowball and convenience sampling methods, the samples in the present dataset are not fully representative of the population in each country. To address this issue, we recommend selection of participants for analysis using a stratified quota sampling method in which data is weighted by the demographics of each country being analysed. For more information on this method, please refer to the original COVIDiSTRESS Global Survey descriptor^1^.

It should be noted that whenever possible, the same methods, variables, and coding were used in this study as in the first COVIDiSTRESS Global Survey study to facilitate comparisons across studies. In addition, the original dataset was lacking data from some regions, so concentrated efforts were made to recruit participants from areas that were underrepresented in the first survey. That said, while some of the scales were used in both studies, the full set of scales administered in this study differed from the COVIDiSTRESS Global Survey in 2020 in order to address the changing landscape of the pandemic (e.g., adding sections about vaccine hesitancy). Because both the scale and the participants differed across COVIDiSTRESS studies, these datasets can be compared, but we recommend caution when combining data across surveys.

## Supplementary information

Supplementary Materials

## Data Availability

The data cleaning notebook and list of variables can be obtained freely here: 10.17605/OSF.IO/36TSD^31^. The data was imported and cleaned using the R software qualtRics, data.table, tidyverse, and multicon. Before analysing the data, it should be noted that invalid cases were excluded and the response options for some variables were recoded to numeric values measuring the degree of agreement (see data cleaning above for details). In some of these options, a neutral value was added to the response options and scored in two different ways. For data quality reasons, we also employed an attention check and filtered data in regard to this check.
