# Author Correction: COVIDiSTRESS diverse dataset on psychological and behavioural outcomes one year into the COVID-19 pandemic

**DOI:** 10.1038/s41597-022-01896-0

**Published:** 2023-01-04

**Authors:** Angélique M. Blackburn, Sara Vestergren, The COVIDiSTRESS II Consortium, Angélique M. Blackburn, Sara Vestergren, Thao P. Tran, Sabrina Stöckli, Siobhán M. Griffin, Evangelos Ntontis, Alma Jeftic, Stavroula Chrona, Gözde Ikizer, Hyemin Han, Taciano L. Milfont, Douglas Parry, Grace Byrne, Mercedes Gómez-López, Alida Acosta, Marta Kowal, Gabriel De Leon, Aranza Gallegos, Miles Perez, Mohamed Abdelrahman, Elayne Ahern, Ahmad Wali Ahmad Yar, Oli Ahmed, Nael H. Alami, Rizwana Amin, Lykke E. Andersen, Bráulio Oliveira Araújo, Norah Aziamin Asongu, Fabian Bartsch, Jozef Bavoľár, Khem Raj Bhatta, Tuba Bircan, Shalani Bita, Hasitha Bombuwala, Tymofii Brik, Huseyin Cakal, Marjolein Caniëls, Marcela Carballo, Nathalia M. Carvalho, Laura Cely, Sophie Chang, Maria Chayinska, Fang-Yu Chen, Brendan Ch’ng, JohnBosco Chika Chukwuorji, Ana Raquel Costa, Vidijah Ligalaba Dalizu, Eliane Deschrijver, İlknur Dilekler Aldemir, Anne M. Doherty, Rianne Doller, Dmitrii Dubrov, Salem Elegbede, Jefferson Elizalde, Eda Ermagan-Caglar, Regina Fernández-Morales, Juan Diego García-Castro, Rebekah Gelpí, Shagofah Ghafori, Ximena Goldberg, Catalina González-Uribe, Harlen Alpízar-Rojas, Christian Andres Palacios Haugestad, Diana Higuera, Kristof Hoorelbeke, Evgeniya Hristova, Barbora Hubená, Hamidul Huq, Keiko Ihaya, Gosith Jayathilake, Enyi Jen, Amaani Jinadasa, Jelena Joksimovic, Pavol Kačmár, Veselina Kadreva, Kalina Kalinova, Huda Anter Abdallah Kandeel, Blerina Kellezi, Sammyh Khan, Maria Kontogianni, Karolina Koszałkowska, Krzysztof Hanusz, David Lacko, Miguel Landa-Blanco, Yookyung Lee, Andreas Lieberoth, Samuel Lins, Liudmila Liutsko, Amanda Londero-Santos, Anne Lundahl Mauritsen, María Andrée Maegli, Patience Magidie, Roji Maharjan, Tsvetelina Makaveeva, Malose Makhubela, María Gálvis Malagón, Sergey Malykh, Salomé Mamede, Samuel Mandillah, Mohammad Sabbir Mansoor, Silvia Mari, Inmaculada Marín-López, Tiago A. Marot, Sandra Martínez, Juma Mauka, Sigrun Marie Moss, Asia Mushtaq, Arian Musliu, Daniel Mususa, Arooj Najmussaqib, Aishath Nasheeda, Ramona Nasr, Natalia Niño Machado, Jean Carlos Natividade, Honest Prosper Ngowi, Carolyne Nyarangi, Charles Ogunbode, Charles Onyutha, K. Padmakumar, Walter Paniagua, Maria Caridad Pena, Martin Pírko, Mayda Portela, Hamidreza Pouretemad, Nikolay Rachev, Muhamad Ratodi, Jason Reifler, Saeid Sadeghi, Harishanth Samuel Sahayanathan, Eva Sanchez, Ella Marie Sandbakken, Dhakal Sandesh, Shrestha Sanjesh, Jana Schrötter, Sabarjah Shanthakumar, Pilleriin Sikka, Konstantina Slaveykova, Anna Studzinska, Fadelia Deby Subandi, Namita Subedi, Gavin Brent Sullivan, Benjamin Tag, Takem Ebangha Agbor Delphine, William Tamayo-Agudelo, Giovanni A. Travaglino, Jarno Tuominen, Tuğba Türk-Kurtça, Matutu Vakai, Tatiana Volkodav, Austin Horng-En Wang Wang, Alphonsus Williams, Charles Wu, Yuki Yamada, Teodora Yaneva, Nicolás Yañez, Yao-Yuan Yeh, Emina Zoletic

**Affiliations:** 1 Texas A&M International University, Laredo, TX USA; 2 Keele University, Keele, England; 3 Department of Psychology and Communications, Texas A&M International University, Laredo, Texas USA; 4 Keele University, Keele, UK; 5 Colorado State University, Fort Collins, Colorado, USA; 6 University of Bern, Bern, Switzerland; 7 Department of Psychology, University of Limerick, Limerick, Ireland; 8 Canterbury Christ Church University, UK & The Open University, Milton Keynes, UK; 9 Peace Research Institute, International Christian University, Tokyo, Japan; 10 Queen’s University Belfast, School of History, Anthropology, Belfast, Northern Ireland; 11 TOBB University of Economics and Technology, Ankara, Turkey; 12 University of Alabama, Tuscaloosa, USA; 13 School of Psychology, University of Waikato, Hamilton, New Zealand; 14 Department of Information Science, Stellenbosch University, Stellenbosch, South Africa; 15 Vrije Universiteit Amsterdam, Amsterdam, Netherlands; 16 Universidad de Córdoba, Córdoba, Spain; 17 Faculty of Health, Universidad Autónoma de Bucaramanga, Bucaramanga, Colombia; 18 Institute of Psychology, University of Wroclaw, Wroclaw, Poland; 19 Texas A&M International University, Laredo, Texas USA; 20 Doha Institute for Graduate Studies, Doha, Qatar; 21 Dublin City University, Dublin, Ireland; 22 Vrije Universiteit Brussel, Brussels, Belgium; 23 University of Chittagong, Chittagong, Bangladesh; 24 Modern University for Business and Science, Beirut, Lebanon; 25 Bahria University Islamabad Campus, Islamabad, Pakistan; 26 Sustainable Development Solutions Network - Bolivia, La Paz, Bolivia; 27 University of Porto, Porto, Portugal; 28 National Centre for Education, Yaounde, Cameroon; 29 Montpellier Buisness School, Montpellier, France; 30 Department of psychology, Faculty of Arts, Pavol Jozef Safarik University in Kosice, Kosice, Slovakia; 31 Master’s program in Counseling Psychology, Tribhuvan University, Kathmandu, Nepal; 32 Tarbiat Modares University, Teheran, Iran; 33 Icare Sustainably international, International, Switzerland; 34 Kyiv School of Economics, Kyiv, Ukraine; 35 Open Universiteit, Heerlen, Netherlands; 36 Departamento de Neurociencia y Aprendizaje, Universidad Católica del Uruguay, Montevideo, Uruguay; 37 Pontifical Catholic University of Rio de Janeiro, Rio de Janeiro, Brazil; 38 Universidad de Los Andes, Bogotá, Colombia; 39 Radboud University, Nijmegen, Netherlands; 40 Pontificia Universidad Católica de Chile, Santiago, Chile; 41 Soochow University, Taiwan, Taipei Taiwan; 42 University of Malaya, Kuala Lumpur, Malaysia; 43 University of Nigeria, Nsukka, Nigeria; 44 The Hill School, Eldoret, Kenya; 45 Ghent University, Ghent, Belgium; 46 University of New South Wales, Kensington, Australia; 47 University College Dublin, Dublin, Ireland; 48 National Research University Higher School of Economics, Moscow, Russian Federation; 49 Universidad del Azuay, Cuenca, Ecuador; 50 The Embassy of Turkey in North Nicosia, Nicosia, Cyprus; 51 Universidad Francisco Marroquin, Guatemala City, Guatemala; 52 Universidad de Costa Rica, Sede de Occidente, Costa Rica, San Ramón, Costa Rica; 53 University of Toronto, Toronto, Canada; 54 ISGlobal (Barcelona Institute for Global Health), Barcelona, Spain; 55 University of Oslo, Norway, Oslo Norway; 56 New Bulgarian University, Sofia, Bulgaria; 57 Ministry of Health, Prague, Czechia; 58 United International University, Dhaka, Bangladesh; 59 Fukuoka Institude Technology, Fukuoka, Japan; 60 University of Belgrade, Faculty of philosophy, Department of psychology, Belgrade, Serbia; 61 Leiden University, Leiden, Netherlands; 62 Department of psychology, Faculty of Arts, Assiut University in Egypt, Asyut, Egypt; 63 Nottingham Trent University, Nottingham, UK; 64 Örebro University, Örebro, Sweden; 65 University of Lodz, Poland, Institute of Psychology, Lodz, Poland; 66 Institute of Psychology Polish Academy of Sciences, Warszawa, Poland; 67 Masaryk University, Brno, Czechia; 68 School of Psychological Sciences, National Autonomous University of Honduras (UNAH), Tegucigalpa, Honduras; 69 The University of Texas at Austin, Austin, Texas USA; 70 Aarhus University, Aarhus, Denmark; 71 Lomonosov MSU, Moscow, Russia; 72 URFU, Ekaterinburg, Russia; 73 Federal University of Rio de Janeiro, Rio de Janeiro, Brazil; 74 Independent Researcher, South Africa, South Africa; 75 Tribhuvan University, Kathmandu, Nepal; 76 Sofia University St. Kliment Ohridski, Sofia, Bulgaria; 77 University of Limpopo, Department of Psychology, Polokwane, South Africa; 78 Russian Academy of Education, Moscow, Russia; 79 AIC RAISE Business Incubator, Rathinam College of Arts and Science India, Coimbatore, India; 80 Trichandra Multiple Campus, Tribhuvan University, Kathmandu, Nepal; 81 University of Milano-Bicocca, Milan, Italy; 82 Rolffortress communications, Kenya, Kenya; 83 National University of Modern Languages, Islamabad, Pakistan; 84 Ludwig Maximilian University, Munich, Germany; 85 SIVIO Institute, Harare, Zimbabwe; 86 Villa College, Maldives, Malé Maldives; 87 Mzumbe University, Dar Es Salaam, Tanzania; 88 University of Nottingham, Nottingham, UK; 89 Department of Civil and Environmental Engineering, Kyambogo University, Uganda, Kampala Uganda; 90 Manipal Institute of Communication, Manipal Academy of Higher Education, Manipal, India; 91 Universidad Rafael Landivar, Guatemala City, Guatemala; 92 Universidad de Las Américas (UDLA), Quito, Ecuador; 93 Mendel University in Brno, Brno, Czechia; 94 Departamento de Psicologia, Universidad Católica de Uruguay, Montevideo, Uruguay; 95 Institute for Cognitive and Brain Sciences Shahid Beheshti University, Tehran, Iran; 96 Faculty of Health and Psychology, State Islamic University of Sunan Ampel, Surabaya, Indonesia; 97 University of Exeter, Exeter, UK; 98 Department of Finance University of Kelaniya, Colombo, Sri Lanka; 99 Oslo New University College, Oslo, Norway; 100 Pavol Jozef Safarik University in Kosice, Kosice, Slovakia; 101 University of Turku, Turku, Finland; 102 University of Skövde, Skövde, Sweden; 103 University of Wellington, Wellington, New Zealand; 104 ICAM, Toulouse, Tolouse France; 105 Sustainable Development Goals Hub, Universitas Indonesia, Depok, Indonesia; 106 International Psychoanalytic University Berlin, Berlin, Germany; 107 University of Melbourne, Melbourne, Australia; 108 National Institute of Cartography Cameroon, Yaounde, Cameroon; 109 Universidad Cooperativa de Colombia, Bogotá, Colombia; 110 Royal Holloway, University of London, London, UK; 111 Univeristy of Turku, Turku, Finland; 112 Trakya University, Edirne, Turkey; 113 North - West University, Potchefstroom, South Africa; 114 Kuban State University, Krasnodar, Russia; 115 University of Nevada, Las Vegas, USA; 116 Sustainable Environmental Solutions-Sweden, Uppsala, Sweden; 117 Purdue University, West Lafayette, Indiana, USA; 118 Kyushu University, Fukuoka, Japan; 119 University of St. Thomas, Houston, USA; 120 Doctoral School of Social Sciencies, University of Warsaw, Warsaw, Poland

**Keywords:** Health policy, Epidemiology, Human behaviour, Developing world, Research data, Health policy, Epidemiology, Human behaviour, Developing world, Research data

Correction to: *Scientific Data* 10.1038/s41597-022-01383-6, published online 21 June 2022.

The original version of this Article contained an error in the spelling of the author Krzysztof Hanusz, which was incorrectly given as Hanusz Krzysztof. This has now been corrected in both the PDF and HTML versions of the Article.

