# Supplementary material for: COVIDiSTRESS diverse dataset on psychological and behavioural outcomes one year into the COVID-19 pandemic
Source: Sci Data. 2022 Jun 21;9:331. doi: 10.1038/s41597-022-01383-6 (PMC9213519; doi:10.1038/s41597-022-01383-6)
Supplement: Supplementary file 1 — Supplementary Materials [file 41597_2022_1383_MOESM1_ESM.pdf]

Table of Contents

Supplementary Table 1 2
Supplementary Table 2 4

**Raw Dataset**		**Cleaned Dataset**	
Residing Country	N	Residing Country	N
Russian Federation	2650	Russian Federation	2260
Japan	2259	Japan	2133
Colombia	1253	Finland	963
Finland	1125	Switzerland	593
Spain	703	Spain	575
Switzerland	686	Colombia	548
Portugal	587	Portugal	484
Honduras	569	Brazil	448
Ireland	547	Honduras	429
Czech Republic	500	Ireland	401
Brazil	493	Norway	376
Norway	453	Czech Republic	365
Italy	429	Slovakia	313
Slovakia	419	Italy	310
Bulgaria	377	Bulgaria	299
Ecuador	376	Ecuador	291
Guatemala	374	Uruguay	288
Uruguay	364	Guatemala	287
Costa Rica	332	Costa Rica	270
Malaysia	327	Kyrgyzstan	254
Kyrgyzstan	304	Ukraine	252
Estonia	301	Estonia	246
Ukraine	295	Malaysia	225
Taiwan	278	Taiwan	221
Pakistan	261	Turkey	200
Sweden	259	Pakistan	157
Turkey	253	Germany	152
Uganda	243	Lebanon	141
Lebanon	190	Uganda	135
Germany	175	Sweden	134
Denmark	163	United Kingdom of Great Britain	134
United Kingdom of Great Britain	155	Denmark	127
Bosnia and Herzegovina	153	Bolivia	115
India	144	United States of America	114
Bolivia	142	Bosnia and Herzegovina	109
United States of America	141	Iran, Islamic Republic of...	90
Poland	113	India	88
Iran, Islamic Republic of...	108	Poland	87
Mexico	95	Mexico	83
Indonesia	73	Greece	54
Kosovo	71	Indonesia	49
Greece	70	Kosovo	48
Nepal	70	Nepal	44
Maldives	59	South Africa	44
Hong Kong (S.A.R.)	59	Hong Kong (S.A.R.)	40
Montenegro	58	Maldives	39
South Africa	53	New Zealand	38
Nigeria	46	Kazakhstan	36
Belgium	45	Montenegro	35
New Zealand	45	Netherlands	35
Kazakhstan	45	Serbia	35
Serbia	44	Belarus	34
Netherlands	43	Belgium	34
France	42	France	32
South Korea	40	Other	446
Belarus	40		
Zimbabwe	38		
Sri Lanka	35		
Republic of Korea	32		
Australia	30		
Canada	30		
Argentina	30		
Republic of Moldova	30		
Other	422		

Supplementary Table 1. Sample size by country for both the raw dataset (N = 20,146) and the cleaned dataset (N = 15740) for countries with more than 200 participants (in yellow) and 30 participants (in green). Note that the cleaned dataset has excluded any participants who failed the attention check or did not otherwise qualify for inclusion.

Surname, Name	Affiliation	Design and Data Collection	Data Cleaning	Analysis	Descriptor	PI
Vestergren, Sara	Keele University	x	x			x
Blackburn, Angélique M.	Department of Psychology and Communications, Texas A&M International University	x	x	x	x	
Tran, Thao P.	Colorado State University	x	x (R codebook)			
Kowal, Marta	Institute of Psychology, University of Wroclaw	x	x			
Stöckli, Sabrina	University of Bern	x	x			
De Leon, Gabriel	Texas A&M International University	x		x		
Sanchez, Eva	Texas A&M International University	x		x		
Aranza Gallegos	Texas A&M International University			x		
Miles Perez	Texas A&M International University			x		
Abdelrahman, Mohamed	Doha Institute for Graduate studies	x				
Acosta, Alida	Faculty of health, Universidad Autónoma de Bucaramanga	x				
Ahern, Elayne	Dublin City University	x				
Ahmad Wali Ahmad Yar	Vrije Universiteit Brussel	x				
Ahmed, Oli	University of Chittagong	x				
Alami, Nael H	Modern University for Business and Science	x				
Amin, Rizwana	Bahria University Islamabad Campus, Pakistan	x				
Andersen, Lykke E.	Sustainable Development Solutions Network - Bolivia	x				
Araújo, Bráulio Oliveira	University of Porto	x				
Bartsch, Fabian	IESEG School of Management	x				
Bavoľár, Jozef	Department of psychology, Faculty of Arts, Pavol Jozef Safarik University in Kosice	x				
Bhatta Khem Raj	Master's program in Counseling Psychology, Tribhuvan University, Nepal	x				
Bircan, Tuba	Vrije Universiteit Brussel	x				
Bita, Shalani	Tarbiat Modares University, Tehran, Iran	x				
Bombuwala, Hasitha	Icare Sustainably international	x				
Brik, Tymofii	Kyiv School of Economics	x				
Byrne, Grace	Vrije Universiteit Amsterdam	x				
Cakal, Huseyin	Keele University	x				
Caniëls, Marjolein	Open Universiteit	x				
Carballo, Marcela	Departamento de Neurociencia y Aprendizaje, Universidad Católica del Uruguay, Montevideo, Uruguay	x				
Carvalho M., Nathalia	Pontifical Catholic University of Rio de Janeiro	x				
Catalina González-Uribe	Universidad de Los Andes	x				
Ćepulić, Dominik-Borna	Catholic University of Croatia	x				
Chang, Sophie	Radboud University	x				
Chayinska, Maria	Pontificia Universidad Católica de Chile	x				
Chen, Fang-Yu	Soochow University, Taiwan	x				
Ch'ng, Brendan	University of Malaya	x				
Chukwuorji, JohnBosco Chika	University of Nigeria, Nsukka.	x				
Costa, Ana Raquel	University of Porto	x				
Dalizu, Vidijah Ligalaba	The Hill School, Eldoret Kenya	x				
Deschrijver, Eliane	Ghent University, University of New South Wales	x				
Diana Higuera	Universidad de Los Andes	x				
Dilekler Aldemir, İlknur	TOBB University of Economics and Technology	x				
Doherty, Anne M	University College Dublin	x				
Doller, Rianne	Icare Sustainably international	x				
Dubrov, Dmitrii	National Research University Higher School of Economics, Moscow, Russia	x				
Elegbede, Salem	icare sustainably international	x				
Elizalde, Jefferson	Universidad del Azuay	x				
Emina Zoletic	Doctoral School of Social Sciencies, University of Warsaw	x				
Ermagan-Caglar, Eda	The Embassy of Turkey in North Nicosia	x				
Fernández-Morales, Regina	Universidad Francisco Marroquin	x				
García-Castro, Juan Diego	Universidad de Costa Rica, Sede de Occidente, Costa Rica	x				
Gelpí, Rebekah	University of Toronto	x				
Ghafori, Shagofah	Vrije Universiteit Brussel	x				
Goldberg, Ximena	ISGlobal (Barcelona Institute for Global Health)	x				
Gómez-López, Mercedes	Universidad de Córdoba, España	x				
Griffin, Siobhán M.	Department of Psychology, University of Limerick, Ireland	x				
Han, Hyemin	University of Alabama	x				
Harlen, Alpízar-Rojas	Universidad de Costa Rica, Sede de Occidente, Costa Rica	x				
Haugestad, Christian Andres Palacios	University of Oslo, Norway	x				
Hoorelbeke, Kristof	Ghent University	x				
Hristova, Evgeniya	New Bulgarian University	x				
Hubená, Barbora	Ministry of Health	x				
Huda Anter Abdallah Kandeel	Department of psychology, Faculty of Arts, Assiut University in Egypt	x				
Huq, Hamidul	United International University, Bangladesh	x				
Ihaya, Keiko	Fukuoka Institude Technology	x				
Ikizer, Gözde	TOBB University of Economics and Technology	x				
Jayathilake, Gosith	Icare Sustainably international	x				
Jeftic, Alma	Peace Research Institute, International Christian University, Tokyo	x				
Jelena Joksimovic	Department of Psychology, University of Belgrade	x				
Jen, Enyi	Radboud University	x				
Jinadasa, Amaani	Icare Sustainably international	x				
Joksimovic, Jelena	University of Belgrade, Faculty of philosophy, Department of psychology	x				
Kačmár, Pavol	Department of psychology, Faculty of Arts, Pavol Jozef Safarik University in Kosice	x				
Kadreva, Veselina	New Bulgarian University	x				
Kalinova, Kalina	Leiden University	x				
Kellezi, Blerina	Nottingham Trent University	x				
Khan, Sammyh	Örebro University	x				
Kontogianni, Maria	Nottingham Trent University	x				
Koszałkowska, Karolina	University of Lodz, Poland, Institute of Psychology	x				
Krzysztof, Hanusz	Institute of Psychology Polish Academy of Sciences	x				
Lacko, David	Masaryk University	x				

Landa-Blanco, Miguel	School of Psychological Sciences, National Autonomous University of Honduras (UNAH)	x				
Laura Cely	Universidad de Los Andes	x				
Lee, Yookyung	The University of Texas at Austin	x				
Lieberoth, Andreas	Aarhus University	x				
Lins, Samuel	University of Porto	x				
Liutsko, Liudmila	1) ISGlobal (Barcelona Institute for Global Health, Spain); 2) Lomonosov MSU (Moscow; Russia); 3) URFU (Ekaterinburg, Russia)	x				
Londero-Santos, Amanda	Federal University of Rio de Janeiro	x				
Maegli, María Andrée	Universidad Francisco Marroquin	x				
Magidie, Patience	Independent Researcher	x				
Maharjan, Roji	Tribhuvan University, Nepal	x				
Makaveeva, Tsvetelina	Sofia University St. Kliment Ohridski	x				
Makhubela, Malose	University of Limpopo, Department of Psychology	x				
Malykh, Sergey	Russian Academy of Education	x				
Mamede, Salomé	University of Porto	x				
Mandillah, Samuel	AIC RAISE Business Incubator, Rathinam College of Arts and Science India	x				
Mansoor, Mohammad Sabbir	Trichandra Multiple Campus, Tribhuvan University, Kathmandu	x				
Mari, Silvia	University of Milano-Bicocca	x				
María Gálvis Malagón	Universidad de Los Andes	x				
Marín-López, Inmaculada	Universidad de Córdoba, España	x				
Marot, Tiago, A.	Pontifical Catholic University of Rio de Janeiro	x				
Mauka, Juma	Rolffortress communications	x				
Mauritsen, Anne Lundahl	Aarhus University	x				
Milfont, Taciano L.	School of Psychology, University of Waikato	x				
Moss, Sigrun Marie	University of Oslo, Norway	x				
Mushtaq, Asia	National University of Modern Languages, Islamabad, Pakistan	x				
Musliu, Arian	Ludwig Maximilian University	x				
Mususa, Daniel	SIVIO Institute	x				
Najmussaqib, Arooj	National University of Modern Languages, Islamabad, Pakistan	x				
Nasheeda, Aishath	Villa College, Maldives	x				
Nasr, Ramona	Modern University for Business and Science	x				
Natalia Niño Machado	Universidad de Los Andes	x				
Natividade, Jean Carlos	Pontifical Catholic University of Rio de Janeiro	x				
Ngowi, Honest Prosper	Mzumbe University	x				
Nicolás Yañez	Universidad de Los Andes	x				
Norah Aziamin Asongu	National Centre for Education	x				
Ntontis, Evangelos	Canterbury Christ Church University, UK & The Open University, UK	x				
Nyarangi, Carolyne	icare sustainably international	x				
Ogunbode, Charles	University of Nottingham, UK	x				
Onyutha, Charles	Department of Civil and Environmental Engineering, Kyambogo University, Uganda	x				
Padmakumar K	Manipal Institute of Communication, Manipal Academy of Higher Education, Manipal, India	x				
Paniagua, Walter	Universidad Rafael Landivar	x				
Parry, Douglas	Department of Information Science, Stellenbosch University	x				
Pena, Maria Caridad	Universidad de Las Américas (UDLA)	x				
Pírko, Martin	Mendel University in Brno	x				
Portela, Mayda	Departamento de Psicologia, Universidad Católica de Uruguay	x				
Pouretemad, Hamidreza	Institute for Cognitive and Brain Sciences Shahid Beheshti University, Tehran, Iran	x				
Rachev, Nikolay	Sofia University St. Kliment Ohridski	x				
Ratodi, Muhamad	faculty of Helath and psychology, State islamic University of Sunan Ampel	x				
Reifler, Jason	University of Exeter	x				
Sadeghi, Saeid	Institute for Cognitive and Brain Sciences Shahid Beheshti University, Tehran, Iran	x				
Sahayanathan, Harishanth Samuel	Department of Finance, University of Kelaniya	x				
Sandbakken, Ella Marie	Bjørknes University College	x				
Sandesh, Dhakal	Tribhuvan University, Nepal	x				
Sandra Martínez	Universidad de Los Andes	x				
Sanjesh, Shrestha	Tribhuvan University	x				
Schrötter, Jana	Pavol Jozef Safarik University in Kosice	x				
Shanthakumar, Sabarjah	Icare Sustainably international	x				
Sikka, Pilleriin	University of Turku; University of Skövde	x				
Slaveykova, Konstantina	University of Wellington	x				
Stavroula Chrona	King's College London, Department of European and International Politics	x				
Studzinska, Anna	University of Economics and Human Sciences in Warsaw & ICAM, Toulouse	x				
Subandi, Fadelia Deby	Sustainable Development Goals Hub, Universitas Indonesia	x				
Subedi, Namita	Tribhuvan University, Nepal	x				
Sullivan, Gavin Brent	International Psychoanalytic University Berlin	x				
Tag, Benjamin	University of Melbourne	x				
Takem Ebangha Agbor Delphine	National Institute of Cartography Cameroon	x				
Tamayo-Agudelo, William	Universidad Cooperativa de Colombia	x				
Travaglino, Giovanni A.	Royal Holloway, University of London	x				
Tuominen, Jarno	Univeristy of Turku	x				
Türk-Kurtça, Tuğba	Trakya University	x				
Vakai, Matutu	North - West University, South Africa	x				
Volkodav, Tatiana	Kuban State University, Krasnodar, Russia	x				
Wang, Austin Horng-En Wang	University of Nevada, Las Vegas	x				
Williams, Alphonsus	Sustainable Environmental Solutions-Sweden	x				
Wu, Charles	Purdue University	x				
Yamada, Yuki	Kyushu University	x				
Yaneva, Teodora	Sofia University St. Kliment Ohridski	x				
Yeh, Yao-Yuan	University of St. Thomas, Houston	x				

Supplementary Table 2. The COVIDiSTRESS Consortium II author contributions. Note that contributors involved in data collection may have been locally supported and/or completed additional local ethics review. The work of Dmitrii Dubrov was supported within the framework of the Basic Research Program at HSE University, RF. The work of Aranza Gallegos and Miles Perez was supported by Texas A & M International University Act on Ideas. Eva Sanchez and Miles Perez were supported by TAMIU Advancing Research and Curriculum Initiative (TAMIU ARC) awarded by the US Department of Education Developing Hispanic-Serving Institutions Program (Award # P031S190304). The work of Angelique M. Blackburn was supported by the Texas A&M International University - University Research Grant 2020-2021.
